# *In vitro* and *in silico* studies reveal antidiabetic properties of arylbenzofurans from the root bark of *Morus mesozygia* Stapf

**DOI:** 10.3389/fphar.2024.1338333

**Published:** 2024-02-23

**Authors:** Katherine Olabanjo Olufolabo, Kai Lüersen, Samuel Ayoolu Oguntimehin, Vaderament-A. Nchiozem-Ngnitedem, Emmanuel Ayodeji Agbebi, Kolade Olatubosun Faloye, Divinah Kwamboka Nyamboki, Gerald Rimbach, Josphat Clement Matasyoh, Bernd Schmidt, Jones Olanrewaju Moody

**Affiliations:** ^1^ Department of Pharmacognosy, Faculty of Pharmacy, Olabisi Onabanjo University, Ago-Iwoye, Nigeria; ^2^ Department of Pharmacognosy, Faculty of Pharmacy, University of Ibadan, Ibadan, Nigeria; ^3^ Institute of Human Nutrition and Food Science, University of Kiel, Kiel, Germany; ^4^ Institut für Chemie, University of Potsdam, Potsdam, Germany; ^5^ Department of Pharmacognosy and Natural Products, College of Pharmacy, Afe Babalola University, Ado-Ekiti, Nigeria; ^6^ Department of Chemistry, Faculty of Science, Obafemi Awolowo University, Ile Ife, Nigeria; ^7^ Department of Chemistry, Faculty of Sciences, Egerton University, Egerton, Kenya

**Keywords:** *Morus mesozygia*, African traditional medicine, root bark extract, arylbenzofuran, α-glucosidase, α-amylase, *in silico* docking

## Abstract

Diabetes remains an important disease worldwide with about 500 million patients globally. In tropical Africa, *Morus mesozygia* is traditionally used in the treatment of diabetes. Biological and phytochemical investigation of the root bark extracts of the plant led to the isolation of a new prenylated arylbenzofuran named 7-(3-hydroxy-3-methylbutyl)moracin M (**1**) and two congeners, moracins P (**2**) and M (**3**). When compared to acarbose (IC_50_ = 486 µM), all the isolated compounds are better inhibitors of α-glucosidase with *in vitro* IC_50_ values of 16.9, 16.6, and 40.9 µM, respectively. However, they were not active against α-amylase. The compounds also demonstrated moderate inhibition of dipeptidyl peptidase-4 (DPP4). Based on *in silico* docking studies, all isolates (**1**, **2**, and **3**) exhibit binding affinities of −8.7, −9.5, and −8.5 kcal/mol, respectively against α-glucosidase enzyme (PDB: 3AJ7). They are stabilized within the α-glucosidase active site through hydrogen bonds, pi interactions, and hydrophobic interactions. This study provides scientific support for the traditional use of *Morus mesozygia* in the treatment of diabetes as well as adding to the repository of α-glucosidase inhibitory agents.

## Introduction

An estimate of people living with diabetes was more than 500 million worldwide as of 2021, and it has been projected that this number will have increased to more than 1.3 billion people by 2050 (Ong et al., 2023). Patients diagnosed with diabetes have high blood glucose levels and are therefore more vulnerable to some of the notorious conditions including stroke, cardiovascular disease, chronic kidney disease, chronic liver disease, infections, and cancer (Lin et al., 2020). Postprandial glucose in the bloodstream is primarily a product of the metabolism of dietary carbohydrates. Two of the enzymes involved in the metabolism of carbohydrates within the digestive tract are α-amylase, which helps break down starch into oligosaccharides, and α-glucosidase, which cleaves oligosaccharides into their monomers (Shanak et al., 2021). Inhibition of these enzymes is one of the strategies used in the treatment of diabetes. Acarbose, for example, is one of the first agents for the treatment of type 2 diabetes being a well-known inhibitor of both α-amylase and α-glucosidase (Irmisch et al., 2019). Another strategy in the treatment of diabetics is the inhibition of dipeptidyl peptidase-4 (DPP4), which functions as a negative regulator of the incretin hormone glucagon-like peptide-1 (GLP-1) by cleaving a terminal dipeptide from the hormone. Blocking DDP4 prevents the inactivation of GLP-1, which results in the enhancement of insulin secretion and regulation of blood glucose levels (Ahrén, 2005).

The often high cost of available treatments coupled with their serious side effects has spurred interest in the search for new antidiabetic agents. Over the past two decades, we have witnessed a proliferation of novel medications designed to lower glucose levels, offering an extensive array of treatment possibilities for individuals dealing with diabetes (Hampp et al., 2014; Dahlén et al., 2022). Even though recent developments in synthesis/medicinal chemistry have played a pivotal role in reaching glycemic objectives, a major drawback of these antidiabetic drugs is the potential for adverse side effects, including gastrointestinal disturbances, weight gain, and hypoglycemia. Additionally, some medications may lose effectiveness over time, leading to the need for increased dosages or alternative treatments (Kothari et al., 2016; Zhu et al., 2021). As a result, it is of utmost importance to underscore the untapped potential of natural compounds derived from plant sources as potent antidiabetic agents. Nature’s pharmacy has long provided Humankind with an array of bioactive molecules that can play a pivotal role in mitigating the effects of diabetes. These plant-derived compounds, often rich in antioxidants and phytochemicals, hold promise in regulating blood sugar levels, improving insulin sensitivity, and reducing the risk of diabetes-related complications.

The plant family Moraceae, which consists of flowering plants, encompasses 38 genera with ∼1100 medicinal plant species, some of which are used traditionally in treating diabetes (Kapche et al., 2009; Tirwomwe et al., 2019). Among the genera found in this family, the genus *Morus*, also known as Mulberry, consists of about 10–16 species widely distributed in subtropical regions of Asia, Africa, and America. One of the species of *Morus* found in West Africa is Black Mulberry or *Morus mesozygia*. The habit of *Morus mesozygia* ranges from a shrub to a small tree, and it is an important member of the tropical African ecosystem with its leaves and fruits serving as food to primates and medicines to humans (Kuete et al., 2009). In African traditional medicine, the aqueous extract of the stem bark is used in the treatment of inflammatory conditions and rheumatic diseases including various types of arthritis (Ariyo et al., 2020) as well as in the treatment of venereal diseases, debility, stomach troubles, pains, infectious diseases, syphilis, asthenias, fever, and malaria (Kuete et al., 2009; Fabien et al., 2012; Oshiomah et al., 2016). Various scientific reports have established the antimicrobial, anticholinesterase, anti-inflammatory, antiplasmodial, cytotoxic, and antioxidant activities of *M. mesozygia* (Kuete et al., 2009; Fabien et al., 2012; Oshiomah et al., 2016; Ariyo et al., 2020). Some flavonoids and arylbenzofuran derivatives isolated from different parts of the plant could be responsible for these pharmacological activities (Kapche et al., 2009; Oshiomah et al., 2016). In a recent investigation, Tirwomwe and co-workers demonstrated the *in vivo* efficacy of *M. mesozygia* leaf extract on the liver and kidneys of alloxan-induced hyperglycemic Wistar rats (Tirwomwe et al., 2019). As part of our continuous search for bioactive secondary metabolites from various Nigerian medicinal plants (Ayoolu et al., 2022), the root bark of *M. mesozygia* harvested in Nigeria was successfully screened in preliminary for their *in vitro* antidiabetic effect. Based on that, a phytochemical study was conducted to identify compounds that may be responsible for the observed activity. Three isolated arylbenzofurans were tested *in vitro* against the antidiabetic target enzymes α-amylase, α-glucosidase and DPP4. *In silico* modeling was used to characterize the interaction of the isolates with the binding pockets of the enzyme α-glucosidase.

## Materials and methods

### General experimental procedures

IR spectra were recorded as ATR-FTIR spectra using a Perkin-Elmer UART TWO FT-IR-spectrometer. The UV/VIS spectra were recorded in high-purity solvents (UVASOl^®^) using the JASCO double-beam photometer (V-630). NMR spectra were recorded in MeOD using a Bruker NEO-500 instrument operating at 500 and 125 MHz for ^1^H and ^13^C{^1^H} NMR, respectively. Spectra referencing was accomplished using the CD_2_ *H*OD solvent peak for ^1^H and the CD_3_OD solvent peak for ^13^C NMR spectra (*δ* = 3.31 and 49.0 for ^1^H and ^13^C{^1^H} signals, respectively). Multiplicities in ^1^H NMR spectra were described using the common descriptors s (singlet), d (doublet), t (triplet), or m (multiplet). High-resolution mass spectra were obtained by EI-TOF (70 eV) using Waters Micromass instruments. Reversed-phase semi-preparative HPLC was performed on Shimadzu LC-20AP pump equipped with DGU-20A5R degassing unit, a Shimadzu SPD-M20A detector, a Shimadzu SIL-20ACHT auto-sampler and a Phenomenex Gemini C_18_ column (10 × 250 mm, 10 μm). Data were recorded and analyzed using LabSolutions software. For column chromatography, Silica gel 60 (0.063–0.2 mm, Macherey-Nagel) was used as solid matrix. TLC was carried out on precoated silica gel 60 plates (0.20 mm). Compounds were visualized under UV light and further by spraying with H_2_SO_4_–EtOH (1:9, v/v). All solvents used were of analytical grade.

### Plant material

Whole *M. mesozygia* plants were collected in Ibadan, Oyo State Nigeria and authenticated in the Herbarium of the Forest Research Institute of Nigeria with herbarium voucher specimen FHI 107677. The root bark of the plants was separated, air-dried under shade and pulverized into powder, before being macerated in 80% aqueous methanol for 72 h, with intermittent stirring. The root bark extract was then filtered, concentrated to dryness under reduced pressure and stored at 4^°^C until further use.

### Extraction and isolation

Three successive extractions were carried out on the powdered air-dried root bark of *M. mesozygia* (3.5 kg) with 80% aqueous MeOH (10 L, 72 h × 3). The extracts were filtered through filter paper (Whatman No. 1) and evaporated under reduced pressure. The crude root bark extract (100 g) was re-suspended in MeOH/H_2_O (1:3, *v*/*v*, 1 L) and successively partitioned with CH_2_Cl_2_ (3 L), and EtOAc (4 L). CH_2_Cl_2_ was used to remove nonpolar compounds in the methanol extract, and subsequent partition with EtOAc was done to remove both moderate and some polar constituents in the extract. All fractions including the aqueous phase were concentrated to dryness at reduced pressure. The EtOAc-soluble fraction (MMRE, 18 g) was further fractionated in silica gel column chromatography using a gradient elution of *n*-hexane/EtOAc with increasing polarity (100:0 to 0:100). About 120 fractions were collected and pooled into 11 subfractions (MMRE_1-11_) based on TLC profiles, which were monitored under an ultraviolet lamp and spraying agents.

Further semi-preparative HPLC on MMRE_9_ using 62% MeOH-H_2_O (0.1% HCOOH) in 22 min at a flow rate of 4 mL/min affording compound **1** (5.4 mg, t_R_ 11.5 min). Subfractions MRRE_1_ and MMRE_2_ were combined and further purified with semi-preparative HPLC using an isocratic elution of 65% MeOH-H_2_O (0.1% HCOOH) in 22 min at a flow rate of 4 mL/min affording compounds **2** (3.2 mg, t_R_ 19.5 min), and **3** (5.4 mg, t_R_ 10.1 min).

7-(3-hydroxy-3-methylbutyl)moracin M *(* ***1*** *)*: brown paste; UV (CH_3_OH) λ_max_ 216, 261, 253, 316, 328 nm; IR (ATR) ν 3351, 2971, 1606, 1424, 1369, 1152, 1051cm^−1^; ^1^H and ^13^C NMR data, see Table 2; HRMS (EI) *m*/*z* 328.1307 [M^+·^] (calcd. for C_19_H_20_O_5_, 328.1311).

Moracin P *(* ***2*** *)*: brown paste; ^1^H NMR (500 MHz, CD_3_OD) *δ* 7.23 (s, 1H), 6.89 (s, 1H), 6.86 (s, 1H), 6.75 (d, *J* = 2.2 Hz, 2H), 6.24 (br t, *J* = 2.2, 1H), 3.79 (dd, *J* = 7.6, 5.3, 1H), 3.12 (dd, *J* = 16.3, 5.3, 1H), 2.83 (dd, *J* = 16.3, 7.6, 1H), 1.36 (s, 3H), 1.28 (s, 3H); ^13^C{^1^H} NMR (125 MHz, CD_3_OD) *δ* 160.0, 156.6, 155.9, 152.6, 133.7, 124.2, 121.8, 117.7, 104.0, 103.7, 101.8, 99.7, 78.2, 70.6, 32.4, 26.0, 21.1; HRMS (EI) *m*/*z* 326.1160 [M^+^] (calcd for C_19_H_18_O_5_, 326.1154).

Moracin M *(* ***3*** *)*: brown paste; ^1^H NMR (500 MHz, CD_3_OD) *δ* 7.35 (d, *J* = 8.4 Hz, 1H), 6.91 (s, 1H), 6.90 (d, *J* = 2.1 Hz, 1H), 6.76 (d, *J* = 2.1 Hz, 2H), 6.73 (dd, *J* = 8.4, 2.1, 1H), 6.24 (m, 1H), ^13^C{^1^H} NMR (125 MHz, CD_3_OD) *δ* 160.0, 157.2, 156.9, 156.1, 133.8, 123.0, 122.0, 113.2, 103.9, 103.5, 102.2, 98.5; HRMS (EI) *m*/*z* 242.0589 [M^+^] (calcd for C_14_H_10_O_4_, 242.0579).

### Antidiabetics study

#### *In-vitro* α-glucosidase inhibition assay

The α-glucosidase activity assay was carried out as described previously (Günther et al., 2021). Briefly, 10 mM stock solutions of the arylbenzofurans (**1—3**) prepared in DMSO were diluted in water. Fifteen µl of these diluted moracin compounds were mixed in 96-well microtest plates (VWR, Darmstadt, Germany) with 105 μL of 0.1 M phosphate buffer, pH 6.8 and 15 μL of a 0.5 U/mL *Saccharomyces cerevisiae* α-glucosidase solution (Sigma-Aldrich, Taufkirchen, Germany). The final arylbenzofurans concentrations range from 0–200 μM, while the final concentration of the extract and fractions ranges from 100–500 μg/mL. The respective solvent controls tested in parallel indicated that enzyme activity was not affected by DMSO concentrations up to 1%. Acarbose was used as a reference inhibitor at the final concentration ranging from 0–10 mM (Günther et al., 2021). Following 5 min pre-incubation at 37°C, the reaction was started with 15 μL of 10 mM p-nitrophenyl-α-D-glucopyranoside (Sigma-Aldrich, Taufkirchen, Germany). After 20 min at 37 °C, 50 μL 2 M Na_2_CO_3_ stop solution was added (VWR, Darmstadt, Germany). The absorbance was measured photometrically at 405 nm (iEMS Reader MF). Five independent experiments were performed in duplicate.

#### *In-vitro* α-amylase inhibition assay

α-Amylase assay was determined using a previously established protocol (Günther et al., 2021). To this end, moracins, extracts, and fraction stocks were diluted with water to give concentrations of 1,000 and 3,000 μg/mL. Of these dilutions, 15 μL were mixed with 35 µL distilled H_2_O and 50 μL of porcine pancreatic α-amylase (2.5 U/mL) in 20 mM sodium phosphate buffer containing 6.7 mM NaCl, pH 6.9. Following 10 min pre-incubation at 25°C, 50 μL 1% starch solution that had been cooked for 15 min in the same buffer was added to start the reaction. After 10 min at 25°C, 100 μL of a color reagent (1% 3,5-dinitrosalicylic acid and 30% sodium potassium tartrate in 0.4 M NaOH, all chemicals from Sigma-Aldrich, Taufkirchen, Germany) were added. The mixture was incubated for an additional 5 min at 100 °C and cooled to room temperature before the absorbance was measured at 540 nm by a microplate reader (iEMS Reader MF, MTX Lab Systems, Helsinki, Finland). Acarbose was used as a reference inhibitor. The percentage inhibition of α-amylase was calculated by using the following equation:

$$\text{Inhibition}\left(\%\right)=\frac{\left[\left(\text{AbC}-{\text{AbC}}_{\text{blank}}\right)-\left(\text{AbS}-{\text{AbS}}_{\text{blank}}\right)\right]}{\left(\text{AbC}-{\text{AbC}}_{\text{blank}}\right)}\times 100$$

AbC, absorbance of the control; AbS, absorbance of the sample.

#### *In-vitro* dipeptidyl peptidase-4 (DPP4) inhibition assay

The DPP4 inhibitor activities of the three moracins were determined by using the DPP4 inhibitor screening kit following the manufacturer’s instructions (abcam, Berlin, Germany). The established DPP4 inhibitor sitagliptin served as the positive inhibitor control, whereas assay buffer was only used as the control for DPP4 enzyme activity and was set to 100%. 30 μL of assay buffer and 10 μL human recombinant DPP4 enzyme were mixed in 96-well microtiter plates with 10 μL of 0.1- or 1-mM moracin solutions, 1 mM sitagliptin, or assay buffer. Following 10 min pre-incubation at 37°C, 50 μL of substrate solution was added to each well. The fluorescence signal (excitation wavelength of 360 nm, emission wavelength of 465 nm) was measured after 30 min incubation at 37°C (Tecan Infinite 200 micro-plate reader). Solvent controls indicated that the DMSO concentrations used did not affect DPP4 activity.

### Molecular docking study

#### Protein and ligand preparation

The 3D crystallographic structure of α-glucosidase (PDB: 3AJ7) was retrieved from the protein data bank (www.rcsb.org). The ions, co-factors, and water molecules were deleted, and a protein bound to the native ligand was retained. Thereafter, the amino acid residues that interacted with the native ligand within 5 Å were identified using the CASTp (Computer Atlas of Surface Topography of proteins) online server for α-glucosidase. Then the native ligand was deleted to obtain a clean protein that was saved in PDB format.

#### Ligand library generation

The 2D structures of compounds **2** and **3** were retrieved from the PubChem online chemical library (https://pubchem.ncbi.nlm.nih.gov/), while **1** was constructed using the MarvinSketch suite, Build 22.12.0–1538, ChemAxon (https://www.chemaxon.com). The energy was minimized under the MMFF94x forcefield at the steepest descent of 0.1. The minimized ligand was thereafter converted from PDB to PDBQT for docking studies (Faloye et al., 2023).

#### Docking procedure validation and molecular docking studies

The molecular docking procedure adopted for these studies was first validated before the experiment was performed. In validating the docking procedure, the native ligand identified at the active site of each enzyme was re-docked by selecting residues within 5 Å, and the RMSD value between the native and re-docked ligand was calculated. Each protein was uploaded on the Autodock Vina built-in interface of the PyRx 0.8 software (Trott and Olson, 2009). Amino acid residues resident within 5 Å were selected and the grid box center and size were adjusted as appropriate. The α-glucosidase enzyme inhibition experiment using target enzyme, 3AJ7 displayed an active center at coordinates of (X = 17.9382, Y = −10.6733, Z = 17.3383). Later, the ligand was loaded in PDBQT format, and docking was initiated at an exhaustiveness of 100. The hydrogen bond, hydrophobic, and π-interactions of the protein-ligand complex obtained from the docking output with the lowest RMSD value and best pose were analyzed with Discovery Studio Visualizer.

#### Drug-likeness study

The Molinspiration tool played a pivotal role in the assessment of the drug-like characteristics of the various drugs. Specifically, it leveraged Lipinski’s Rule of Five (RO5) as a foundational framework to gauge the potential bioavailability and pharmacokinetics of substances when taken orally. The absorption, distribution, metabolism, and excretion (ADME) of all compounds were assessed using the Molinspiration online database (https://molinspiration.com/) to predict their physicochemical properties.

#### IC_50_ calculation and statistical analyses

IC_50_ values for the purified compounds and the reference inhibitor acarbose were calculated by using the online tool (https://www.aatbio.com/tools/ic50-calculator). GraphPad Prism, version 5.0 was used for the statistical analysis of data, which were expressed as mean ± standard deviation. Multiple comparisons of IC_50_ values were carried out by using one-way ANOVA followed by Tukey’s test. For the DPP4 inhibition results, a one-way ANOVA followed by Dunnett’s post-hoc test was conducted to compare the means of treatment groups to that of the control group without inhibitor.

## Results

### Screening of root bark extract and fractions of *Morus mesozygia* for α-glucosidase and α-amylase inhibition

Medicinal plants are key elements in the treatment of various diseases, particularly among the less developing nations of the world. From one culture to another, different parts of *M*. *mesozygia* are employed in the treatment of different diseases including diabetes. The anti-diabetic properties of different parts (leaves, stem bark, and rook bark) of *M. mesozygia* were therefore evaluated. It was observed that the aqueous MeOH extract of the root bark inhibited *S*. *cerevisiae* α-glucosidase and porcine pancreatic α-amylase with IC_50_ of 157.5 and 136.6 μg/mL, respectively (Table 1). The root bark extract was further partitioned into CH_2_Cl_2_, EtOAc, and H_2_O and afterward tested for their enzyme inhibitory activity. The EtOAc fraction had the highest activity against α-glucosidase and α-amylase enzymes with IC_50_ of 223.5 and 290.5 μg/mL, respectively, which interestingly was significantly better than the standard drug used, acarbose (Table 1). Ethyl acetate is a moderately polar solvent used in the extraction of moderately polar compounds such as polyphenols including flavonoids, arylbenzofurans, tannins, and some saponins (Rahmawati et al., 2023). These compounds are well known to possess various bioactive activities and accordingly could be responsible for the observed enzyme-inhibitory activities of this fraction. Based on this preliminary results, the EtOAc fraction of *M*. *mesozygia* attracted our attention with the aim of identifying the active ingredients responsible for these activities.

**TABLE 1:** Half-maximal inhibitory concentrations (IC_50_) of the methanolic extract from *Morus mesozygia* root bark and fractions obtained from it towards α-glucosidase and α-amylase enzyme activities.

	IC_50_ (µg/mL)	
*M. mesozygia*	α-Glucosidase	α-Amylase
Root bark extract	157.5 ± 0.4^a^	136.6 ± 1.5^a^
Dichloromethane fraction	336.1 ± 0.6^b^	586.4 ± 0.7^b^
Ethylacetate fraction	223.5 ± 0.2^c^	290.5 ± 0.7^c^
Aqueous fraction	227.8 ± 0.9^c^	589.1 ± 0.9^d^
Acarbose	343.3 ± 1.6^d^	472.3 ± 0.1^e^

Values expressed as IC_50_ ± standard error mean (n = 3). Values with the same superscripts in columns are not significantly different, while those with different superscripts are (*p* < 0.05; multiple comparison by using one-way ANOVA, followed by Tukey’s test).

### Phytochemical study of the ethyl acetate fraction

**FIGURE 1:**
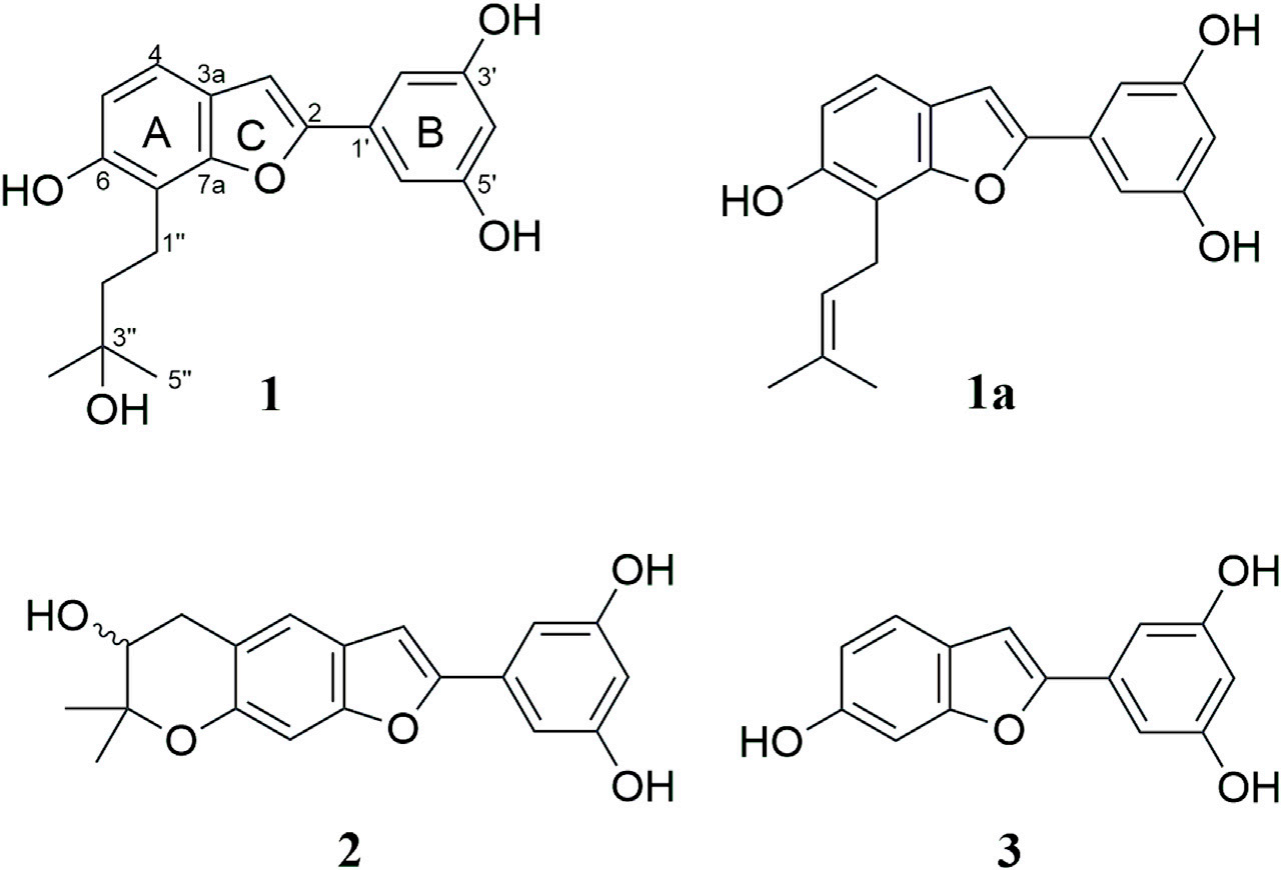
Structures of arybenzofurans isolated from the rook bark of *Morus mesozygia*.

Multiple-step chromatography separation of the EtOAc fraction led to the isolation and characterization of three secondary metabolites. Out of these, a new arylbenzofuran derivative (**1**) alongside two biosynthetically related compounds, moracin P (**2**) (Ferrari et al., 1998; Nguyen et al., 2009; Sivaraman et al., 2019), and moracin M (**3**) (Hyun et al., 2007; Sivaraman et al., 2019) were isolated (Figure 1). Their molecular structure was established based on 1D and 2D-NMR, high-resolution mass spectrometry as well as by comparison with those reported in the literature. The characterization of the new compound is discussed below.

Compound **1** was isolated as a brown paste. The chemical structure (Figure 1) was confirmed as C_19_H_20_O_5_ (10 indices of hydrogen deficiency (IHD)) based on HREIMS, which displayed a molecular ion peak at *m*/*z* 328.1307 [M^+·^] (calcd. for C_19_H_20_O_5_, 328.1311). Its IR absorption bands unveiled the presence of free hydroxy and aryl functionalities. The ^1^H NMR (Table 2) disclosed five signals in the aromatic region, out of which a signal observed at *δ*_H_ 6.90 (s, 1H) together with those from ^13^C NMR [(*δ*_C_ 102.5 (C-3), 122.9 (C-3a), 155.9 (C-2), 155.9 (C-7a))] spectrum were characteristic of an arylbenzofuran (10 IHD) skeleton (Ni et al., 2009; Wu et al., 2019). The remaining four aromatic protons were assigned to A-ring, AB-type spin system with resonances at *δ*_H_ 7.18 (d, *J* = 8.3 Hz, 1H) and 6.73 (d, *J* = 8.3 Hz, 1H), while signals at *δ*_H_ 6.79 (d, *J* = 2.2 Hz, 2H) and 6.24 (t, *J* = 2.2 Hz, 1H) were ascribed to a trisubstituted benzene B-ring. The ^13^C NMR (Table 2) spectrum displayed 17 signals, out of which two are caused by two symmetry equivalent carbons, as indicated by the HSQC- and HMBC-spectra (cross peaks to signals integrating for two protons in the ^1^H-NMR spectrum). Out of the 17 signals in the ^13^C-NMR spectrum, seven are from quaternary carbons (six of which are oxygenated), five from C-H-carbons, two from CH_2_-groups, and one signal results from two methyl groups (cross peak to a singlet integrating for 6 protons in the HSQC). The aforementioned data combined with 2D NMR (^1^H–^1^H COSY, HSQC and HMBC) data suggested that compound **1** bears a C-7 prenylated arylbenzofuran similar to moracin S (**1a**) (Kapche et al., 2009), except for the signals derived from the prenyl group. The lipophilic side chain in **1** had 18 atomic mass unit (amu) higher than that observed in moracin S (**1a**), indicating the addition of one molecule of H_2_O. With A-C rings of the arylbenzofuran secured, it was evident that the prenyl side part in moracin S underwent selective hydrolysis giving rise to the corresponding alcohol as shown in **1**. The C-3ʹʹ resonance of **1** was shifted upfield by Δ*δ*_C_ 60.3 as compared with that of moracin S (**1a**), suggesting that C-3ʹʹ is a tertiary alcohol **1**. The placement of the -OH group at C-3ʹʹ was further supported by HMBC correlations from H-4ʹʹ/5ʹʹ (*δ*_H_ 1.34) with *δ*_C_ 71.8 (C-3ʹʹ), H-2ʹʹ (*δ*_H_ 1.87) with *δ*_C_ 71.8 (C-3ʹʹ), and H-1ʹʹ (*δ*_H_ 2.99) with *δ*_C_ 71.8 (C-3ʹʹ), Table 2. An important correlation was observed in the ^1^H–^1^H COSY spectrum, showing the presence of a spin system among signals at *δ*_H_ 2.99 (H-1″) and 1.87 (H-2″). Just like in moracin S, the aliphatic side chain in **1** was placed at C-7 based on HMBC correlations between H-1ʹʹ (*δ*_H_ 2.99) with *δ*_C_ 43.8 (C-2ʹʹ), 71.8 (C-3ʹʹ), 153.9 (C-6), 113.6 (C-7), and 155.9 (C-7a). The systematic name of compound **1** is 5-(6-hydroxy-7-(3-hydroxy-3-methylbutyl)benzofuran-2-yl)benzene-1,3-diol, but we suggest the trivial name 7-(3-hydroxy-3-methylbutyl)moracin M). A SciFinder search revealed that compound **1** has been assigned a CAS-number and that the compound is offered commercially as a screening compound by two suppliers. However, no publications describing either a chemical synthesis or the isolation from a natural source are available, and no analytical or spectroscopical data were published.

**TABLE 2:** ^13^C (125 MHz) and ^1^H (500 MHz) NMR spectroscopic data of compound **1** in CD_3_OD.

Position	1		
	*δ*_C_, _Type_	*δ*_H_, mult. (*J* in *Hz*)	HMBC (H→C)
2	155.9, C		
3	102.5, CH	6.90 s	C-2, C-3a
3a	122.9, C		
4	118.9, CH	7.18 d (8.3)	C-3, C-6, C-7a
5	113.1, CH	6.73 d (8.3)	C-3a, C-6, C-7
6	153.9, C		
7	113.6, C		
7a	155.9, C		
1ʹ	134.0, C		
2ʹ/6ʹ	103.9, CH	6.79 d (2.2)	C-2, C-2ʹ/6ʹ, C-4ʹ, C-3ʹ/5ʹ
3ʹ/5ʹ	159.9, C		
4ʹ	103.4, CH	6.24 t (2.2)	C-2ʹ/6ʹ, C-3ʹ/5ʹ
1ʹʹ	19.8, CH_2_	2.99 m	C-2ʹʹ, C-3ʹʹ, C-6, C-7, C-7a
2ʹʹ	43.8, CH_2_	1.87 m	C-1ʹʹ, C-3ʹʹ, C-4′ʹ/5′ʹ, C-7
3ʹʹ	71.8, C		
4ʹʹ/5ʹʹ	29.1, CH_3_	1.34 s	C-2ʹʹ, C-3ʹʹ, C-4′ʹ/5′ʹ

### Antidiabetic assessment of arylbenzofurans (**1–3**) isolated from the EtOAc fraction of Morus mesozygia

Arylbenzofurans (**1—3**) were evaluated for their *in vitro* inhibitory activity against α-glucosidase from *S*. *cerevisiae* and porcine α-amylase. Compounds **1** and **2** were found to be potent α-glucosidase inhibitors *in vitro* with IC_50_ values of 16.9 and 16.6 µM, respectively. Moracin M (**3**) was slightly less efficient having a mean IC_50_ value of 40.9 µM. Interestingly, these compounds (**1—3**) were about 10 to 30-fold more potent than the reference drug acarbose (Table 3). On the contrary, when tested on α-amylase at concentrations up to 300 μM, the moracins (**1—3**) had virtually no impact on the *in vitro* enzyme activity. In turn, the reference inhibitor acarbose inhibited the α-amylase activity with an IC_50_ value of 8.7 µM (Table 3).

**TABLE 3:** *In vitro* α-glucosidase and α-amylase inhibitory activities of 2-arylbenzofuran derivatives (**1—3**).

Samples	α-glucosidase IC_50_ [µM] (range)	α-amylase IC_50_ [µM] (range)
**1**	16.9^a^ (10.8—21.8)	>300
**2**	16.6^a^ (11.4—26.9)	>300
**3**	40.9^a^ (18.4—60.3)	>300
Acarbose	486^b^ (399–624)	8.7 (8.5—8.9)

IC_50_ values were determined in n = 5 independent experiments performed in duplicate for α-glucosidase and in n = 2 independent experiments performed in duplicate for α-amylase. The IC_50_ values of compound one to three for α-glucosidase were significantly lower than the value for the reference inhibitor acarbose (*p* < 0.001, one-way ANOVA, with Tukey’s multiple comparisons test). Values sharing the same superscript are not statistically different.

**FIGURE 2:**
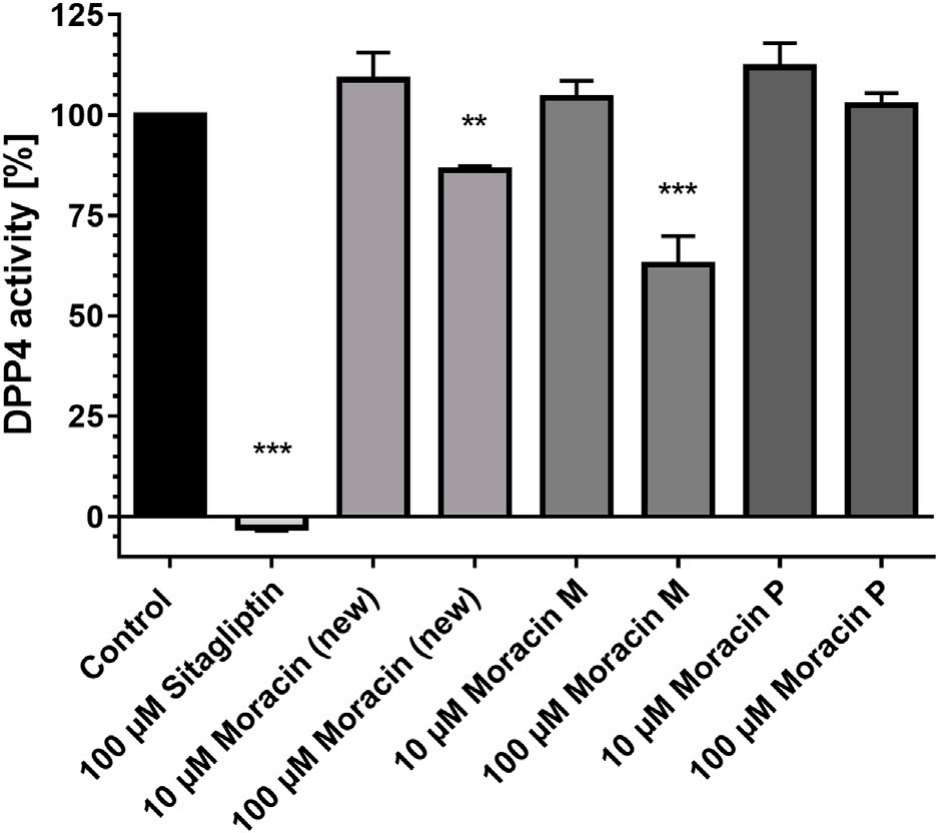
Compound **1** (new moracin) and moracin M (**3**) exhibit moderate inhibitory effects on the *in-vitro* activity of human dipeptidyl peptidase-4 (DPP4) enzyme activity. DPP4 assays were carried out in the presence of the indicated substances. The percentage values of remaining DPP4 enzyme activities in comparison to the control are shown. Results are mean values of n = 3 experiments performed in duplicate. Error bars indicate the standard deviation. ***p < 0.001, **p < 0.01, one-way ANOVA with posthoc multiple comparison tests of Dunnett.

When assayed against dipeptidyl peptidase 4 (DPP4) *in vitro,* compounds **1** and **3** were moderate inhibitors of DPP4. At 100 μM, the new arylbenzofuran derivative (**1**) displayed 15% inhibitory activity against DPP4, whereas moracin M (**3**) showed a superior effect resulting in a residual peptidase activity of 62.7% compared to control. At 10-fold dilution, none of the tested moracins showed any impact on DPP4 (Figure 2).

### Computational study of arylbenzofurans (**1—3**) against α-glucosidase

The potent α-glucosidase inhibitory effect of tested compounds (**1—3**) compared to the standard drug acarbose prompted us to further investigate their *in silico* and physicochemical properties. Therefore, molecular docking was performed using AutoDock software to evaluate the binding ability of the tested compounds with α-glucosidase (PDB: 3AJ7), while the calculation of drug-likeness properties was performed using Molinspiration online database.

**FIGURE 3:**
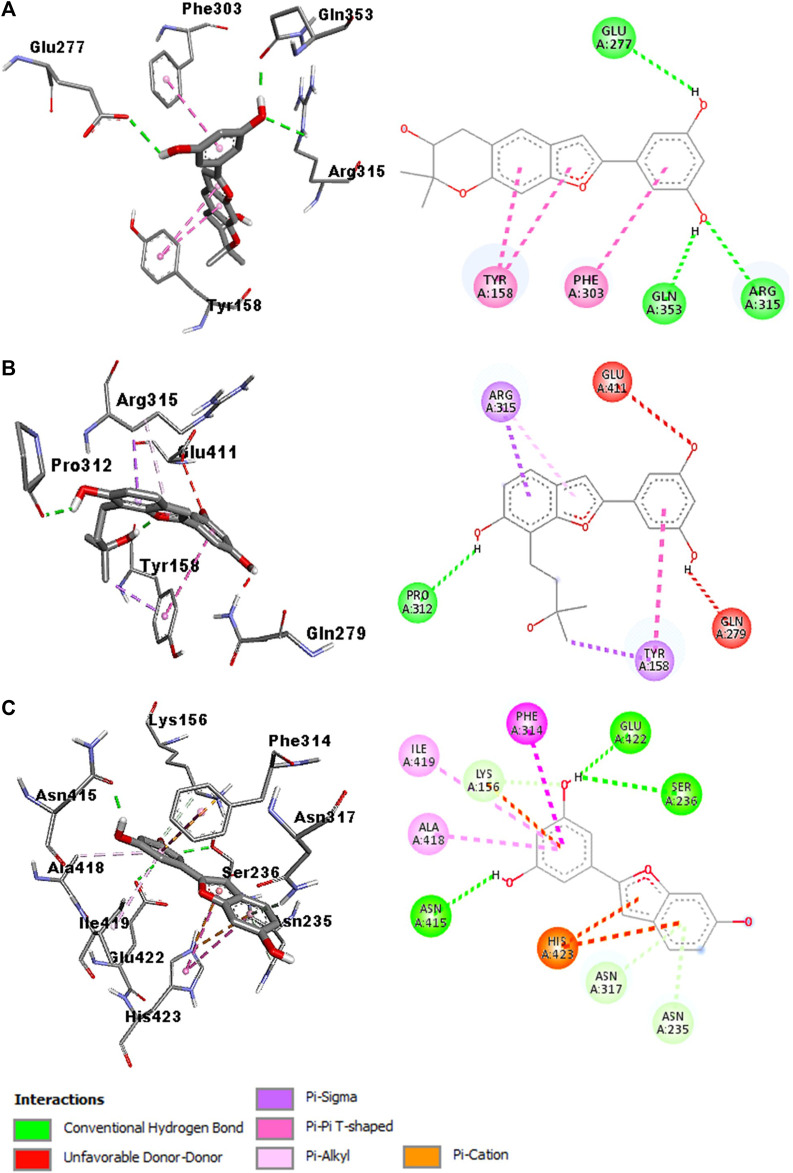
Interaction diagram of compound **2** (moracin P)-3AJ7 **(A)**, compound **1**-3AJ7 **(B)**, and compound **3** (moracin M)-3AJ7 **(C)**

The molecular docking studies identified the three arylbenzofurans as potential α-glucosidase inhibitors after the binding affinity of acarbose (−8.3 kcal/mol) was set as the cut-off point. Compound **2** (Moracin P) was selected as the best binder with a binding affinity of −9.5 kcal/mol. The oxygen and hydrogen atoms on the moracin P moiety participated in three hydrogen bond interactions with Glu277 at 2.51 Å, Arg315 at 2.78 Å, and Glu353 at 1.98 Å. The phytochemical further attained stability by establishing hydrophobic and pi-interactions with Tyr158 and Phe303 (Figure 3A).

Compound **1** was identified as the second-best binder with a binding affinity of −8.7 kcal/mol. The oxygen atom in moracin moiety established four hydrogen bond interactions with Pro312 at 2.18 Å. Also, the ligand was stabilized at the binding pocket of the α-glucosidase enzyme by participating in hydrophobic and pi-interactions with Tyr158 and Arg315 (Figure 3B).

Compound **3** (Moracin M) was ranked as the third-best binder with a binding affinity of −8.5 kcal/mol. The hydrogen atoms on the moracin M moiety established three hydrogen bond interactions with Ser236 at 2.73 Å, Asn415 at 1.86 Å, and Glu422 at 2.31 Å. The phytochemical also participated in hydrophobic interactions with Phe314, Ala418, Ile419 and His423. Furthermore, the stability of moracin M was strengthened by forming pi-interactions with Phe314, Ala418, Ile419, and His423 (Figure 3C). *In silico* studies also predicted that the compounds meet all the criteria for a suitable drug candidate. They have a low molecular weight (<500 g/mol) and do not violate Lipinski’s rule (Table 4).

**TABLE 4:** Physicochemical properties of compounds (**1—3**).

Samples	%ABS	TPSA (Å^2^)	*n*-ROTB	Molecular weight	miLogP	*n*-OHNH donors	*n*-ON acceptors	Lipinski’s violations
criteria				<500	≤5	<5	<10	≤1
**1**	76.6	94.05	4	328.4	3.92	4	5	0
**2**	80.3	83.06	1	326.4	3.32	3	5	0
**3**	83.5	73.82	1	242.2	2.65	3	4	0

%ABS: percentage of absorption = 109—(0.345*TPSA); TPSA: topological polar surface area; *n*-ROTB: number of rotatable bonds; miLogP: logarithm of compound partition coefficient between *n*-octanol and water; *n*-OHNH: number of hydrogen bond donors; *n*-ON: number of hydrogen bond acceptors.

## Discussion

Africa is well known for its biodiversity and scientific investigation has revealed that numerous plant species are well known for the treatment and management of diabetes mellitus (Afolayan and Sunmonu, 2010; Neelesh et al., 2010; Ndip et al., 2013; Mohammed et al., 2014). Furthermore, various compounds sourced from African ethnomedicinal plants have demonstrated potential antidiabetic activity in laboratory settings (*in vitro*) or in living organisms (*in vivo*). In the present study, we have demonstrated that three moracins isolated from the medicinal plant African mulberry *M. mesozygia* are efficient α-glucosidase inhibitors, which is in good accordance with the traditional knowledge of *M*. *mesozygia* root for the treatment of diabetes. Similarly, a literature survey unveiled that various tissues (leaf/root bark) of the related species *Morus alba* showed *in vivo* antidiabetic effect (Mohammed et al., 2014). Various arylbenzofuran including moracins P (**2**) and M (**3**) have been reported from both plants as well as from other *Morus* species, therefore, can be considered as chemotaxonomic markers of plants belonging to this genus. From the ethnopharmacological standpoint, moracin P (**2**) isolated from the Chinese crude drug Sang-Bai-Pi (*Morus* root bark) showed marginal inhibitory effect against protein tyrosine phosphatase 1B (PTP1B) (Cui et al., 2006). In antiviral assay, moracin P (**2**) showed potent inhibitory activity (IC_50_ = 42.9 µM) against chronic hepatitis C virus (HCV) cell, while moracin M (**3**) lacking the additional ring (pyrone ring) was less potent (IC_50_ > 100 µM) (Hyun et al., 2007). Further, moracin P (**2**) in concentration dependent manner (0.3 nM - 1 µM), displayed inhibitory potency against pro-inflammatory mediator focusing on the inflammation-associated transcription factor (NF-κB) in breast cell line. Additionally, the cytoprotective effects of moracin P (**2**) against TRAIL-induced cellular damage in HaCaT cells was also observed (Besse et al., 2020). More recently, moracin P (**2**) (EC_50_ = 8.8 µM) exhibited cytoprotective effect against doxorubicin-induced myocardial toxicity in H9c2 cells (Zheng et al., 2017). Moracin M (**3**) which can be considered as a precursor in the biosynthesis of various arylbenzofurans has received much attention from the scientific communities. This compound displayed a broad spectrum of biological activities such as *in vitro* and *in vivo* anti-inflammatory (Lee et al., 2016; Guo et al., 2018; Lee et al., 2020), antimicrobial (Kuete et al., 2009), and phosphodiesterase-4 inhibitor (Chen et al., 2012). Furthermore, *in vivo* hypoglycemic effects of moracin M (**3**) from the root bark of *Morus alba* has been reported (Zhang et al., 2009). Although the pharmacological effects of related compounds moracins P (**2**) and M (**3**) have extensively been investigated, to the best of our knowledge the chemistry as well as the antidiabetic effect of the new compound (**1**) is reported here for the first time. *In vitro* α-glucosidase assays revealed that all arylbenzofurans displayed inhibitory activity, whereby compounds **1** and **2** being the most potent agents with almost identical IC_50_ values of about 17 µM. A key structure-activity-relationship (SAR) among these compounds suggests that the presence of a modified prenyl group at C-7 in **1** or an extra double bond equivalent with 2,2-dimethylpyran moiety derived from the modified prenyl fragment at C5 and the hydroxyl group at C6 in **2** are minimum requirements for α-glycosidase inhibitory activity. Intriguingly, moracin M (**3**) lacking these features was ∼2.4-fold less potent than **1** and **2**. These results are superimposable with previous research findings, which showed that prenylation increase the lipophilicity of arylbenzofuran, thereby led to stronger inhibitory activities compared to their non-prenylated counterpart (Hoang et al., 2009; Zhang et al., 2014). In addition, to examine the mechanism of action through which all three compounds (**1**, **2**, and **3**) inhibited α-glucosidase enzyme, the molecular docking studies was performed thereby providing relevant insights into the interactions established with the active amino acid residues at the binding site of the enzyme.

It is worth noting that the physicochemical properties of the three plant-based metabolites (**1—3**) follow and satisfy Lipinski’s rule of 5 (Lipinski, 2004; Lipinski et al., 2012). All tested compounds showed %ABS (Percentage of Absorption) ranging from 76% (compound **1**) to 83% (compound **2**) which indicated that a significant portion of the drug reaches the bloodstream (Zhao et al., 2002). Unlike acarbose, whose low %ABS value suggests poor absorption, the isolated moracins may therefore not only target gastrointestinal enzymes (α-glucosidase and α-amylase), but also additional endogenous steps of glucose metabolism such as the DDP4 enzyme activity examined here. It is therefore certainly worthwhile to investigate the effect of isolated arylbenzofurans on other antidiabetic targets such as sodium-dependent glucose transporters 1 and 2, glucagon-like peptide 1, glucose transporter GLUT4 or insulin secretion (Cavin et al., 2016; Günther et al., 2021). Above all, this finding complements previous studies, which demonstrated that arylbenzofuran such as moracins P (**2**) and M (**3**) have a broad spectrum of activities including antidiabetic effects.

Of note, the *Morus* root bark extract exhibited also an α-amylase inhibitory activity that was predominantly present in the ethyl acetate fraction, but that was found to be not related to the purified moracins. Various metabolites including flavonoids, 2-arylbenzofuran, stilbenes, Diels–Alder-type adducts, and alkaloids could be responsible for this activity. Hence, *Morus* extract comprised further putatively intriguing compounds with antidiabetic activity that should be investigated in future studies.

In conclusion, this study helped establish the antidiabetic properties of the root bark extract of *M*. *mesozygia* in the treatment of diabetes. A new arylbenzofuran derivative was identified to be a better inhibitor of α-glucosidase than acarbose. The observed selectivity of the novel compound, as well as the two previously known compounds, in relation to α-glucosidase, suggests that they may not possess undesirable adverse effects. Consequently, these compounds exhibit potential as promising antidiabetic drug candidates. However, more studies are needed to evaluate its bioavailability, efficiency, and safety *in vivo* models and ultimately in humans.

## Data Availability

The original contributions presented in the study are included in the article/Supplementary Material, further inquiries can be directed to the corresponding authors.
