# Supplementary material for: In vitro and in silico studies reveal antidiabetic properties of arylbenzofurans from the root bark of Morus mesozygia Stapf
Source: Front Pharmacol. 2024 Feb 23;15:1338333. doi: 10.3389/fphar.2024.1338333 (PMC10935558; doi:10.3389/fphar.2024.1338333)

*Supplementary Material*

# Antidiabetic in vitro and *in Silico* Study of Arylbenzofuran from Root Bark of *Morus mesozygia* Stapf

**Katherine Olabanjo Olufolabo[1,2†], Kai Lüersen[3*†], Samuel Ayoolu Oguntimehin[2], Vaderament-A. Nchiozem-Ngnitedem[4], Emmanuel Ayodeji Agbebi[5], Kolade Olatubosun Faloye[6], Divinah Kwamboka Nyamboki[7], Gerald Rimbach[3], Josphat Clement Matasyoh[7], Bernd Schmidt[4], Jones Olanrewaju Moody[2*]**

[1]*Department of Pharmacognosy, Faculty of Pharmacy, Olabisi Onabanjo University, Ago-Iwoye, Ogun State, Nigeria.*

[2]*Department of Pharmacognosy, Faculty of Pharmacy, University of Ibadan, Ibadan, Nigeria*

[3]*Institute of Human Nutrition and Food Science, University of Kiel, Kiel, Germany*

[4]*Institut für Chemie, University of Potsdam, Potsdam-Golm, Germany*

[5]*Department of Pharmacognosy and Natural Products, College of Pharmacy, Afe Babalola University, Ado-Ekiti, Nigeria*

[6]*Department of Chemistry, Faculty of Science, Obafemi Awolowo University, Ile Ife, Nigeria*

[7]*Department of Chemistry, Faculty of Sciences, Egerton University, Egerton, Kenya*

*** Correspondence:**
Corresponding Author
luersen@foodsci.uni-kiel.de
jo.moody@mail.ui.edu.ng

[†] These authors share first authorship

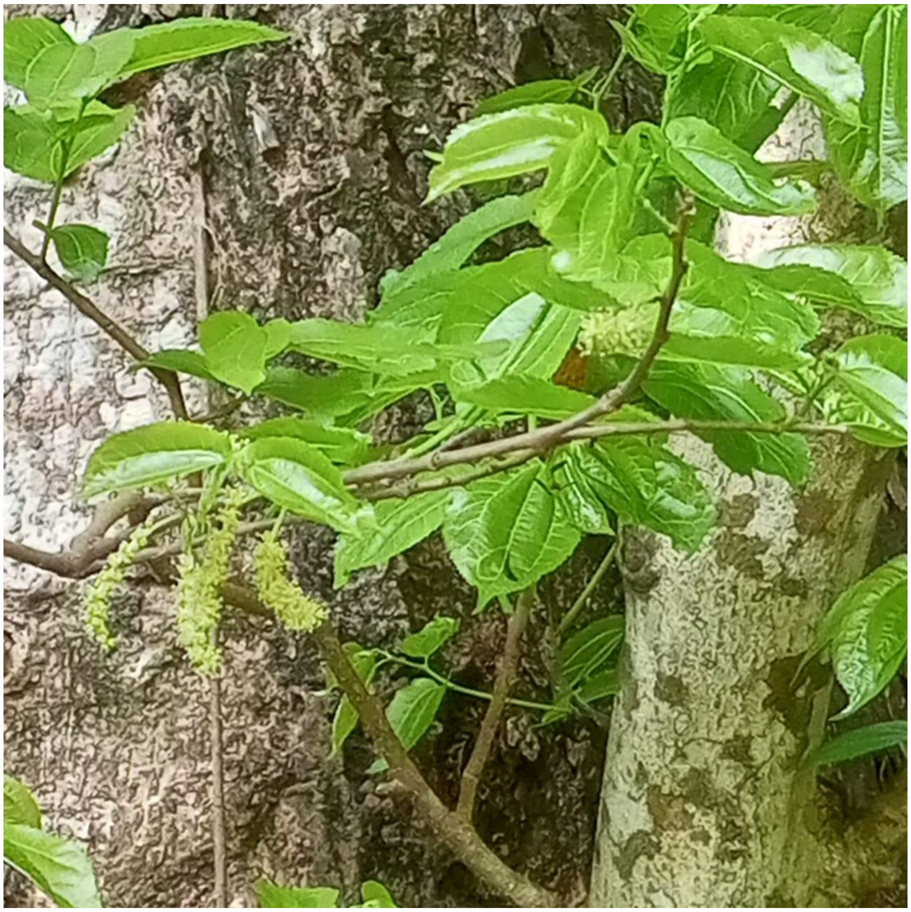

**Figure S1**: *Morus mesozygia* growing *in situ (original picture taken in the botanical garden of Ibadan, Nigeria)*

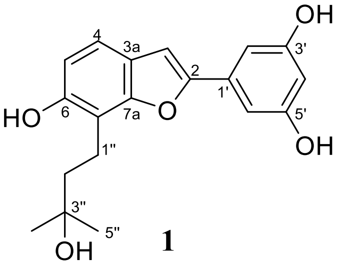


**1**

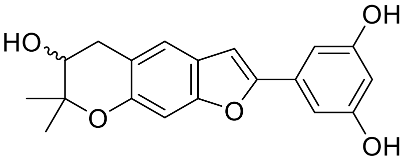


**2**

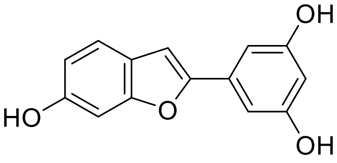


**3**

**Figure S2**: Structures of arybenzofurans isolated from the rook bark of *Morus mesozygia*

**Table S1**: $^{13}C$ (125 MHz) and $^{1}H$ (500 MHz) NMR spectroscopic data of compound **1** in $CD_3OD$

| | **1** | | |
|---|---|---|---|
| Position | $\delta_C$, Type | $\delta_H$, mult. (*J* in *Hz*) | HMBC (H→C) |
| 2 | 155.9, C | | |
| 3 | 102.5, CH | 6.90 s | C-2, C-3a |
| 3a | 122.9, C | | |
| 4 | 118.9, CH | 7.18 d (8.3) | C-3, C-6, C-7a |
| 5 | 113.1, CH | 6.73 d (8.3) | C-3a, C-6, C-7 |
| 6 | 153.9, C | | |
| 7 | 113.6, C | | |
| 7a | 155.9, C | | |
| 1′ | 134.0, C | | |
| 2′/6′ | 103.9, CH | 6.79 d (2.2) | C-2, C-2′/6′, C-4′, C-3′/5′ |
| 3′/5′ | 159.9, C | | |
| 4′ | 103.4, CH | 6.24 t (2.2) | C-2′/6′, C-3′/5′ |
| 1″ | 19.8, CH2 | 2.99 m | C-2″, C-3″, C-6, C-7, C-7a |
| 2″ | 43.8, $CH_2$ | 1.87 m | C-1″, C-3″, C-4″/5″, C-7 |
| 3″ | 71.8, C | | |
| 4″/5″ | 29.1, $CH_3$ | 1.34 s | C-2″, C-3″, C-4″/5″ |

CAS-number of 1: **2184940-21-2**; compound **1** has been assigned a CAS-number, the compound is offered commercially as a screening compound by two suppliers, but no publications describing either chemical synthesis or the isolation from a natural source are available.

*Moracin P (**2**)*: Brown paste; $^{1}H$ NMR (500 MHz, $CD_3OD$) $\delta$ 7.23 (s, 1H), 6.89 (s, 1H), 6.86 (s, 1H), 6.75 (d, *J* = 2.2 Hz, 2H), 6.24 (br t, *J* = 2.2, 1H), 3.79 (dd, *J* = 7.6, 5.3, 1H), 3.12 (dd, *J* = 16.3, 5.3, 1H), 2.83 (dd, *J* = 16.3, 7.6, 1H), 1.36 (s, 3H), 1.28 (s, 3H); $^{13}C\{^{1}H\}$ NMR (125 MHz, $CD_3OD$) $\delta$ 160.0, 156.6, 155.9, 152.6, 133.7, 124.2, 121.8, 117.7, 104.0, 103.7, 101.8, 99.7, 78.2, 70.6, 32.4, 26.0, 21.1; HREIMS *m*/*z* 326.1160 [$M^+$] (calcd for $C_{19}H_{18}O_5$, 326.1154). Analytical data match those previously described in the literature. (Total synthesis: Sivaraman, A., Harmalkar, D. S., Kang, J., Choi, Y., & Lee, K. (2019). A protecting group-free divergent synthesis of natural benzofurans via one-pot synthesis of 2-bromo-6-hydroxybenzofurans. Organic & Biomolecular Chemistry, 17(8), 2153-2161; Isolation from natural source: Lee et al. (2011). Inhibitory Effect of 2-Arylbenzofurans

from the Mori Cortex Radicis (Moraceae) on Oxygen Glucose Deprivation (OGD)-induced Cell Death of SH-SY5Y Cells, Arch. Pharm. Res., 34(8), 1373-1380.)); NMR-data match those described in these papers well.

*Moracin M (**3**)*: Brown paste; $^{1}H$ NMR (500 MHz, $CD_3OD$) $\delta$ 7.35 (d, *J* = 8.4 Hz, 1H), 6.91 (s, 1H), 6.90 (d, *J* = 2.1 Hz, 1H), 6.76 (d, *J* = 2.1 Hz, 2H), 6.73 (dd, *J* = 8.4, 2.1, 1H), 6.25-6.23 (m, 1H), $^{13}C\{^{1}H\}$ NMR (125 MHz, $CD_3OD$) $\delta$ 160.0, 157.2, 156.9, 156.1, 133.8, 123.0, 122.0, 113.2, 103.9, 103.5, 102.2, 98.5; HREIMS *m/z* 242.0589 [$M^+$] (calcd for $C_{14}H_{10}O_4$, 242.0579).

Ref.: a) Total synthesis: A. Sivaram et al., Org. Biomol. Chem., 2019, 17, 2153-2161 (DOI: 10.1039/c8ob03102a); b) Isolation from natural source: Jeong et al., (2009), Tyrosinase Inhibitory Polyphenols from Roots of *Morus lhou*, J. Agric. Food Chem., 57(4), 1195-1203. NMR-data match those described in these papers well.

$^{1}$H NMR (500 MHz, $CD_3OD$) of compound **1**

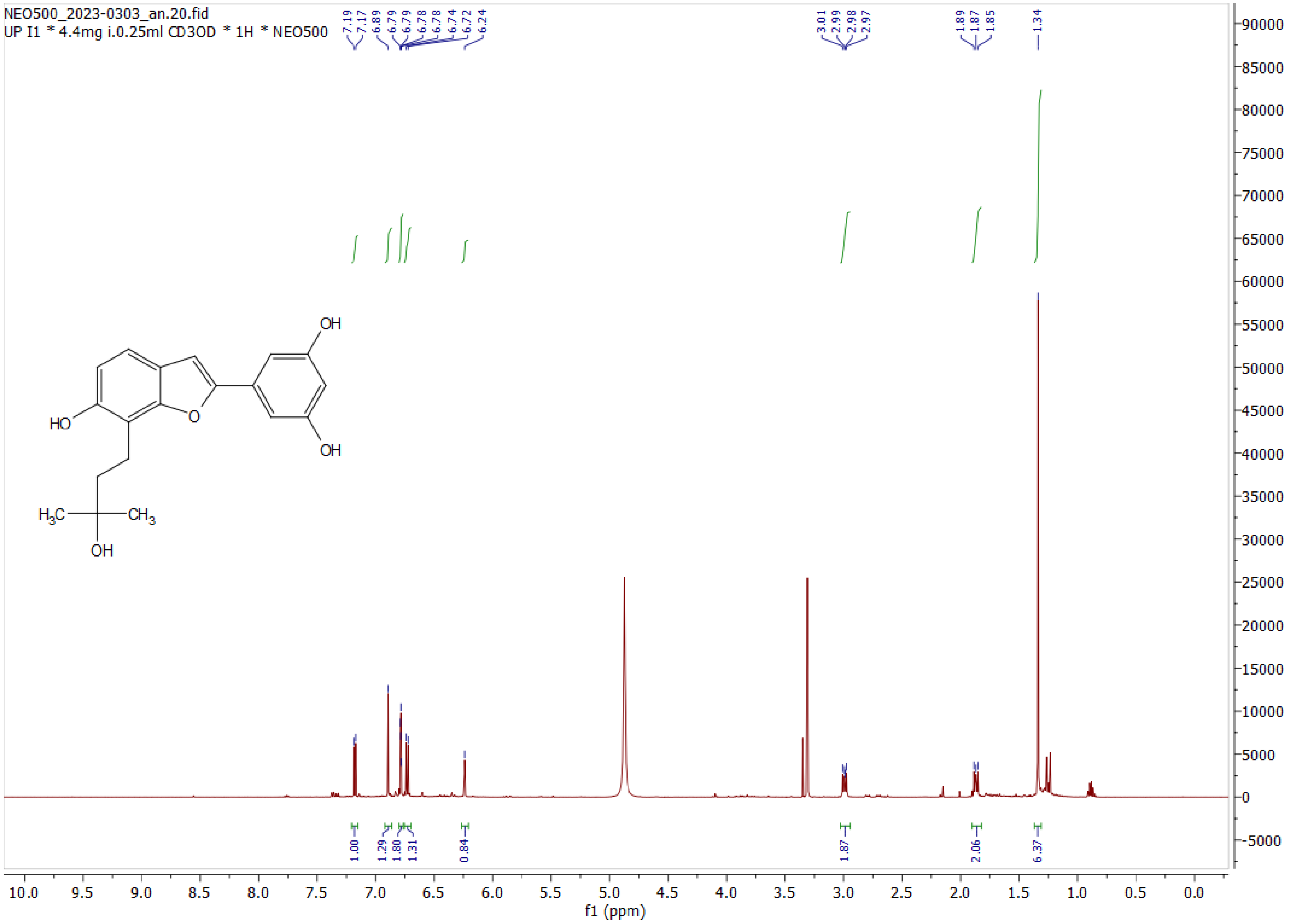

$^{13}$C NMR (125 MHz, $CD_3OD$) of compound **1**

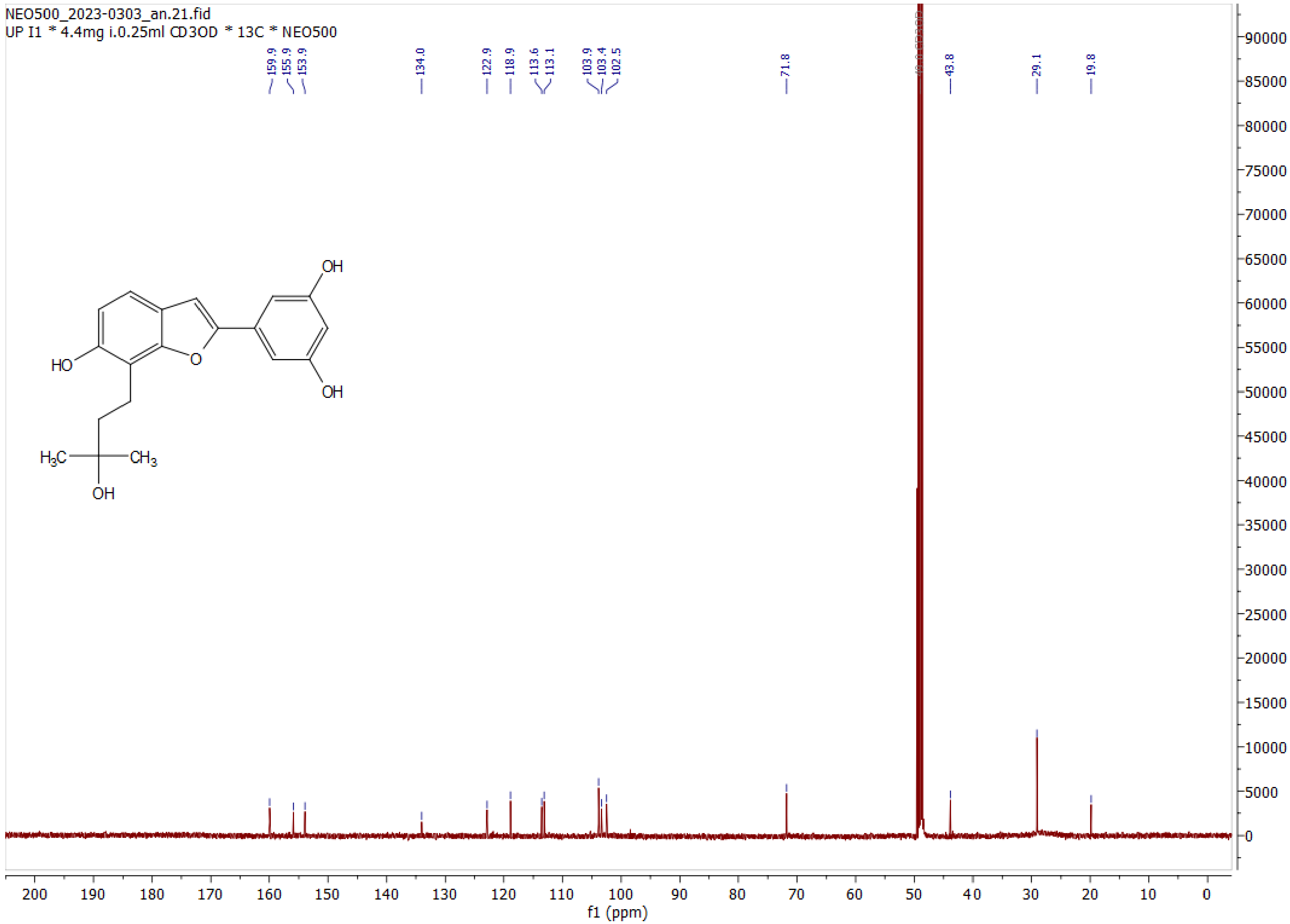

COSY (500 MHz, $CD_3OD$) of compound **1**

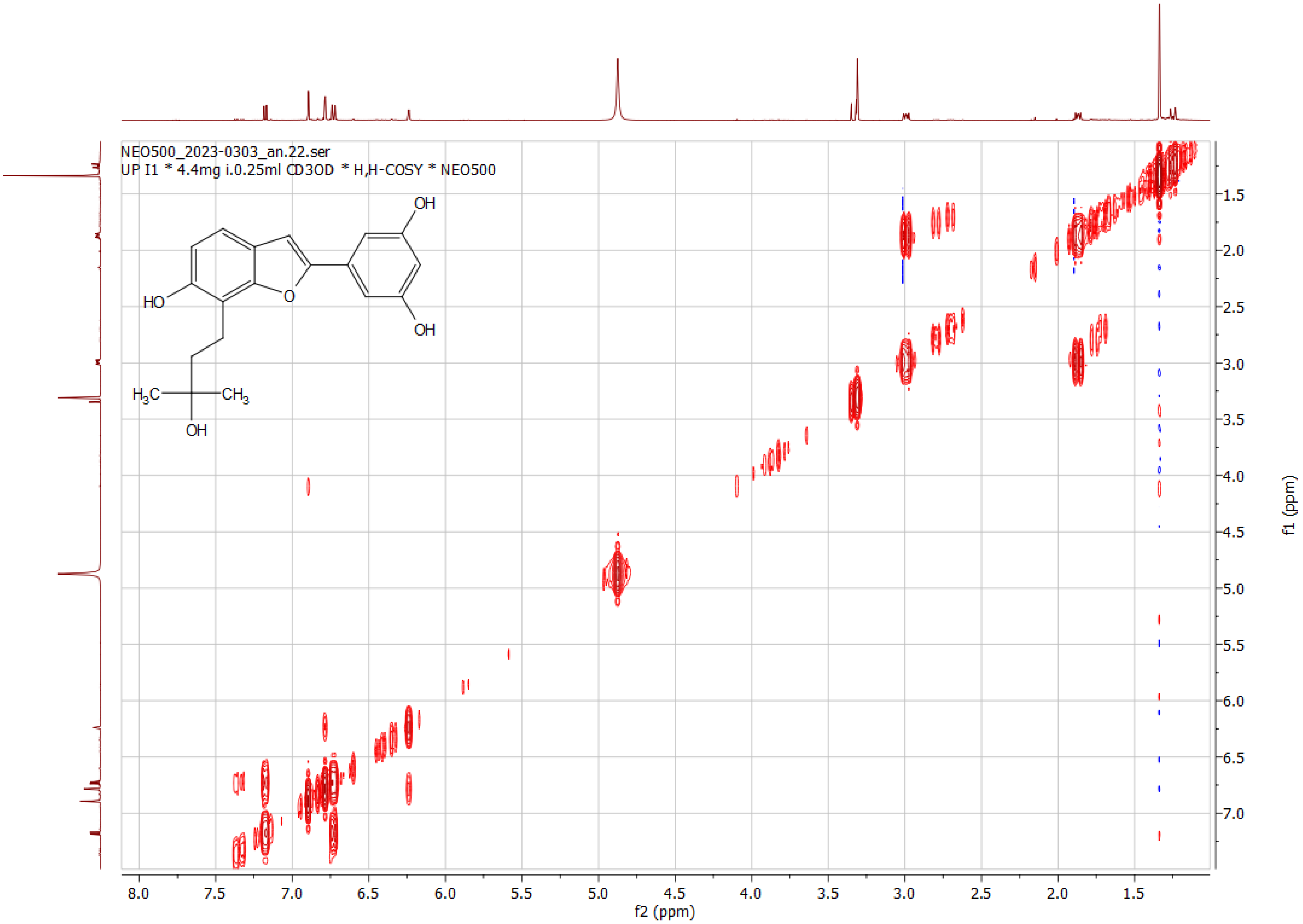

HSQC (500/125 MHz, $CD_3OD$) of compound **1**

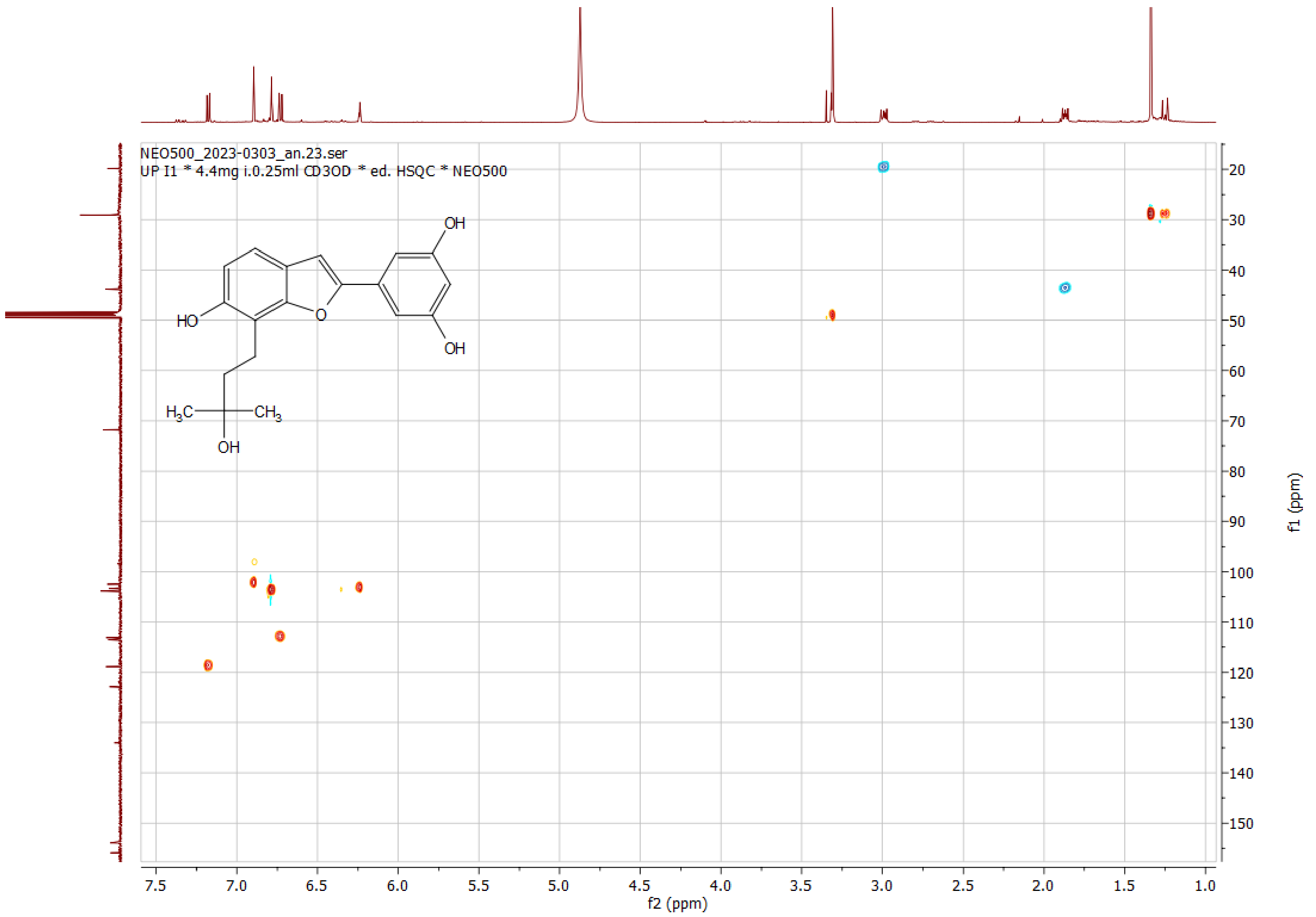

HMBC (500/125 MHz, $CD_3OD$) of compound **1**

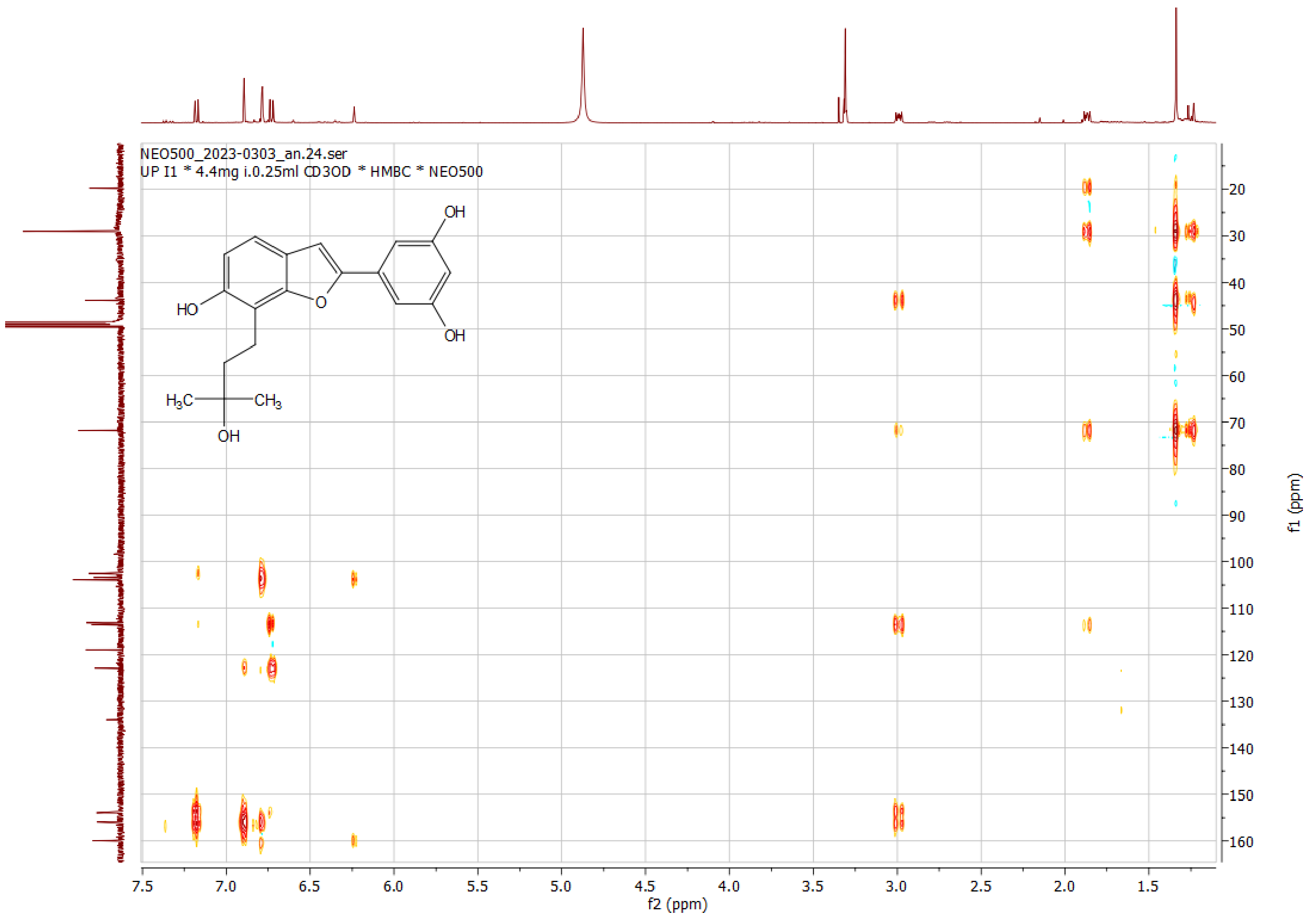

$^{1}H$ NMR (500 MHz, $CD_3OD$) of moracin P (**2**)

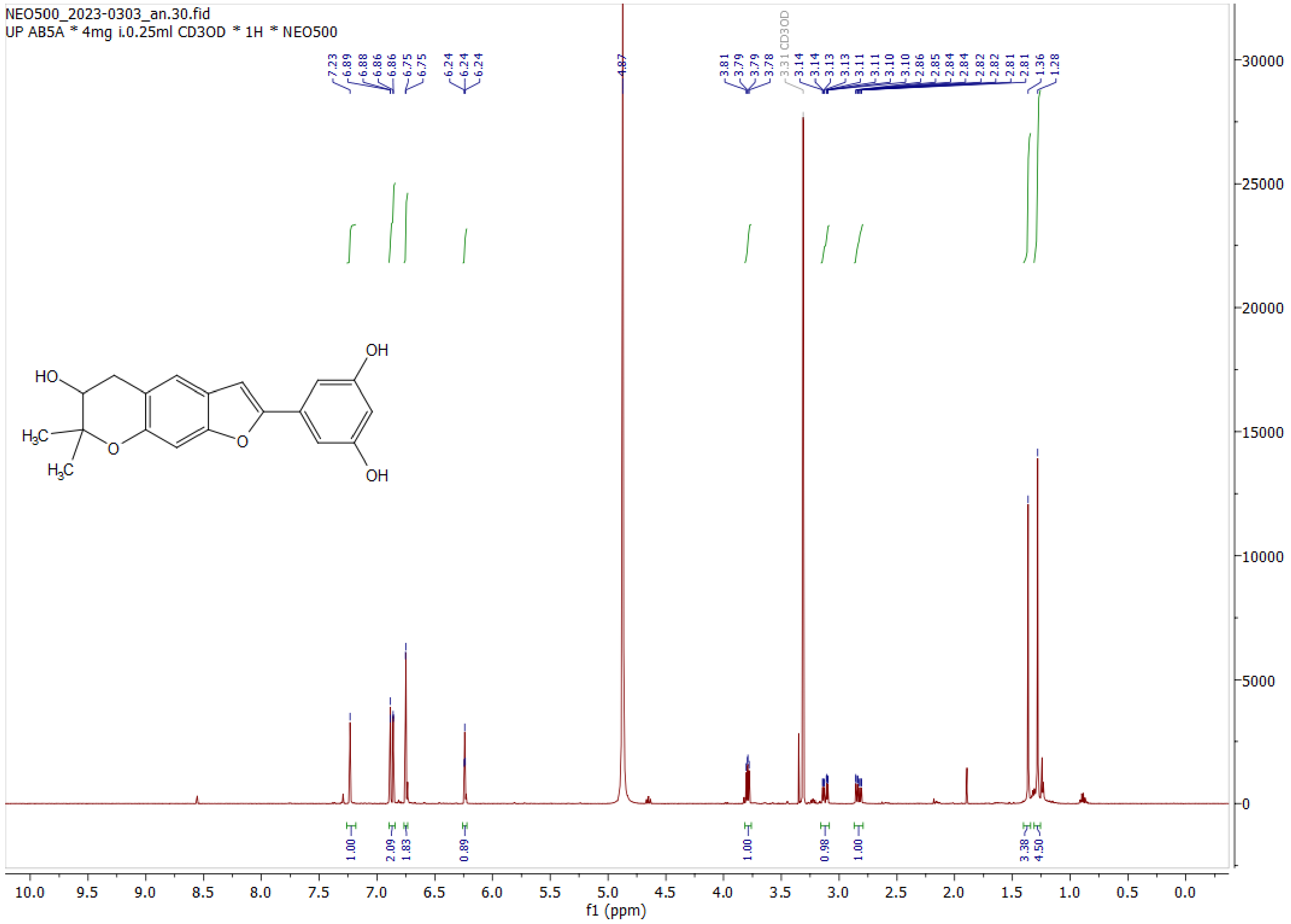

$^{13}$C NMR (125 MHz, $CD_3OD$) of moracin P (**2**)

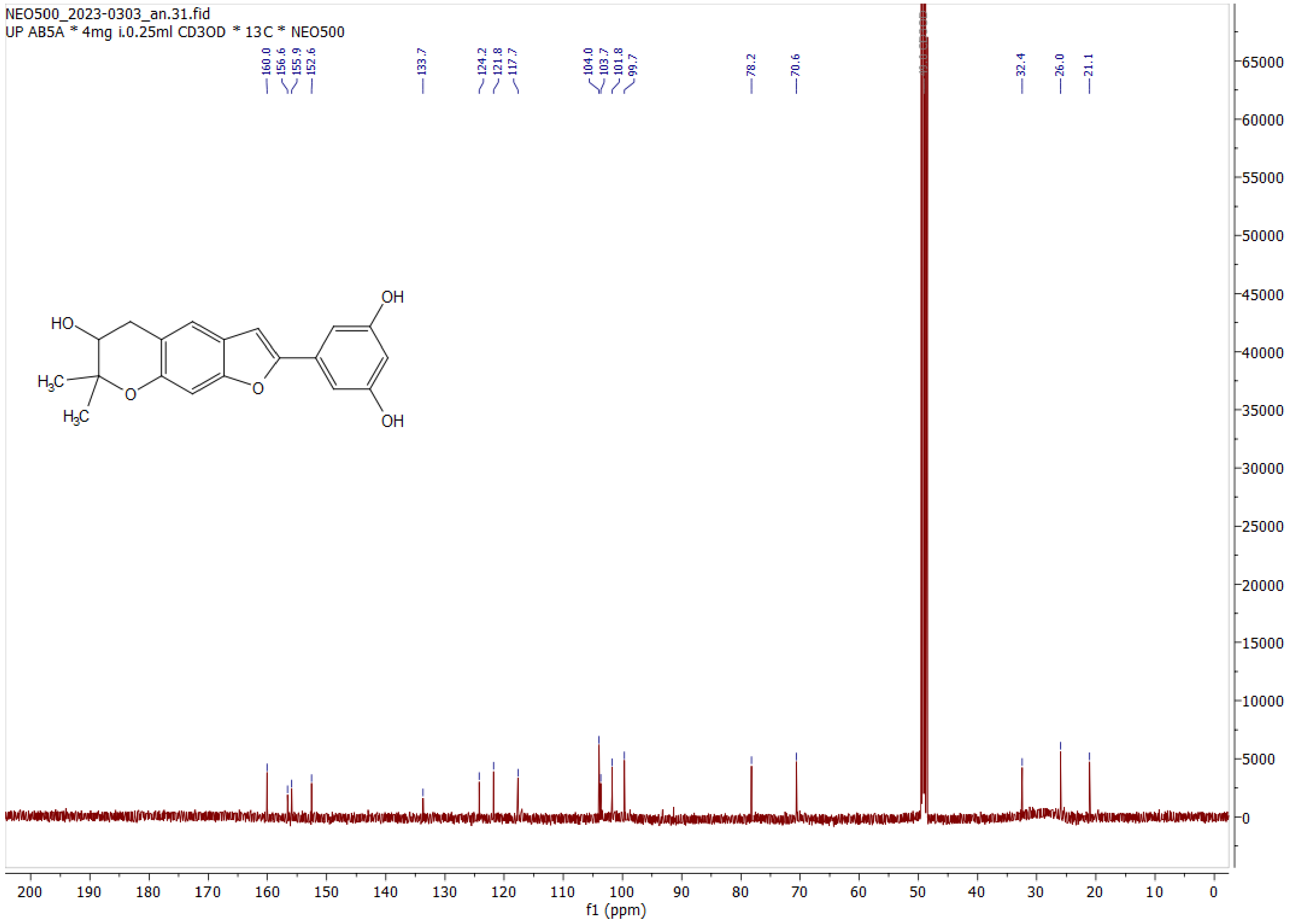

COSY (500 MHz, $CD_3OD$) of moracin P (**2**)

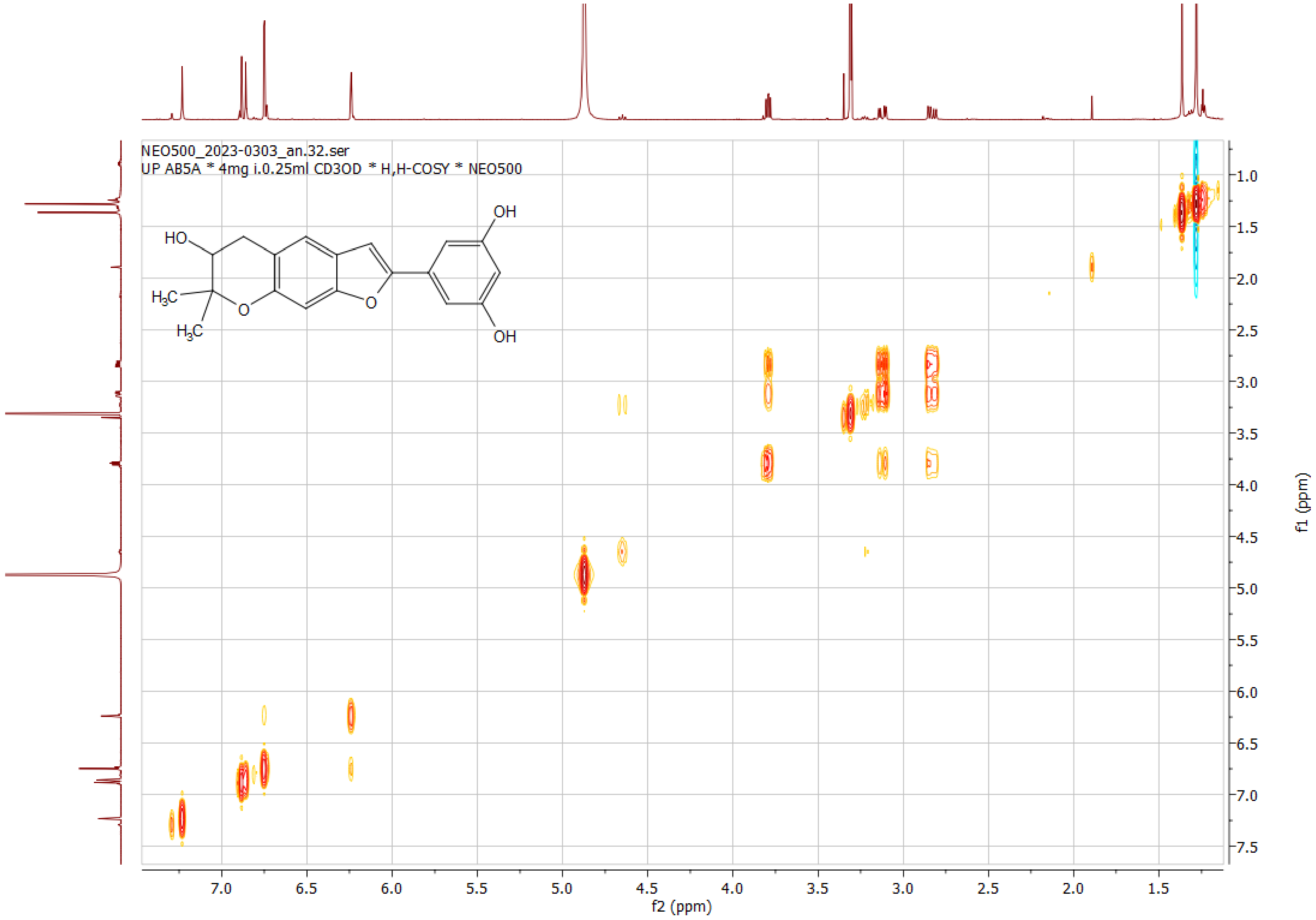

HSQC (500/125 MHz, $CD_3OD$) of moracin P (**2**)

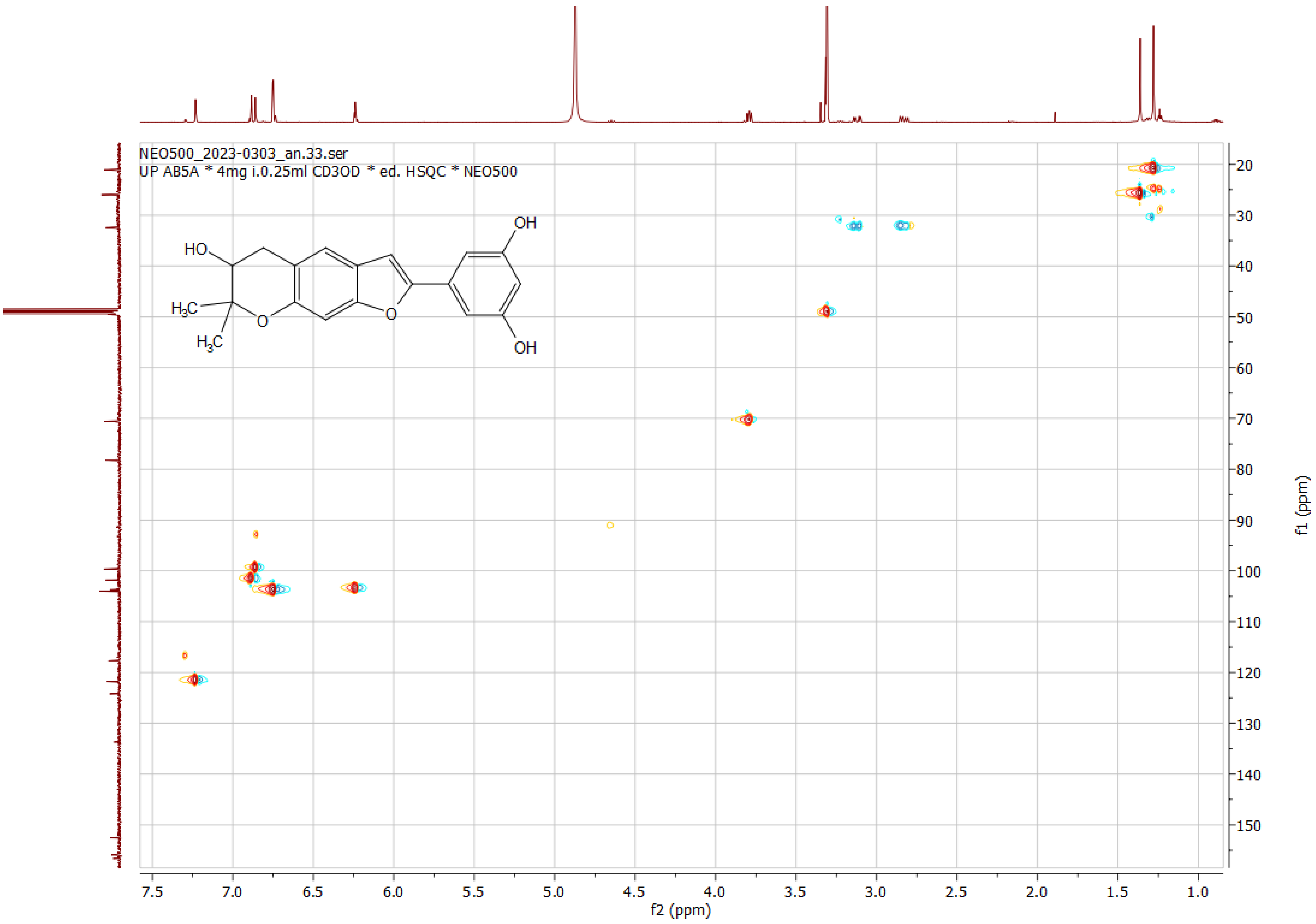

HMBC (500/125 MHz, $CD_3OD$) of moracin P (**2**)

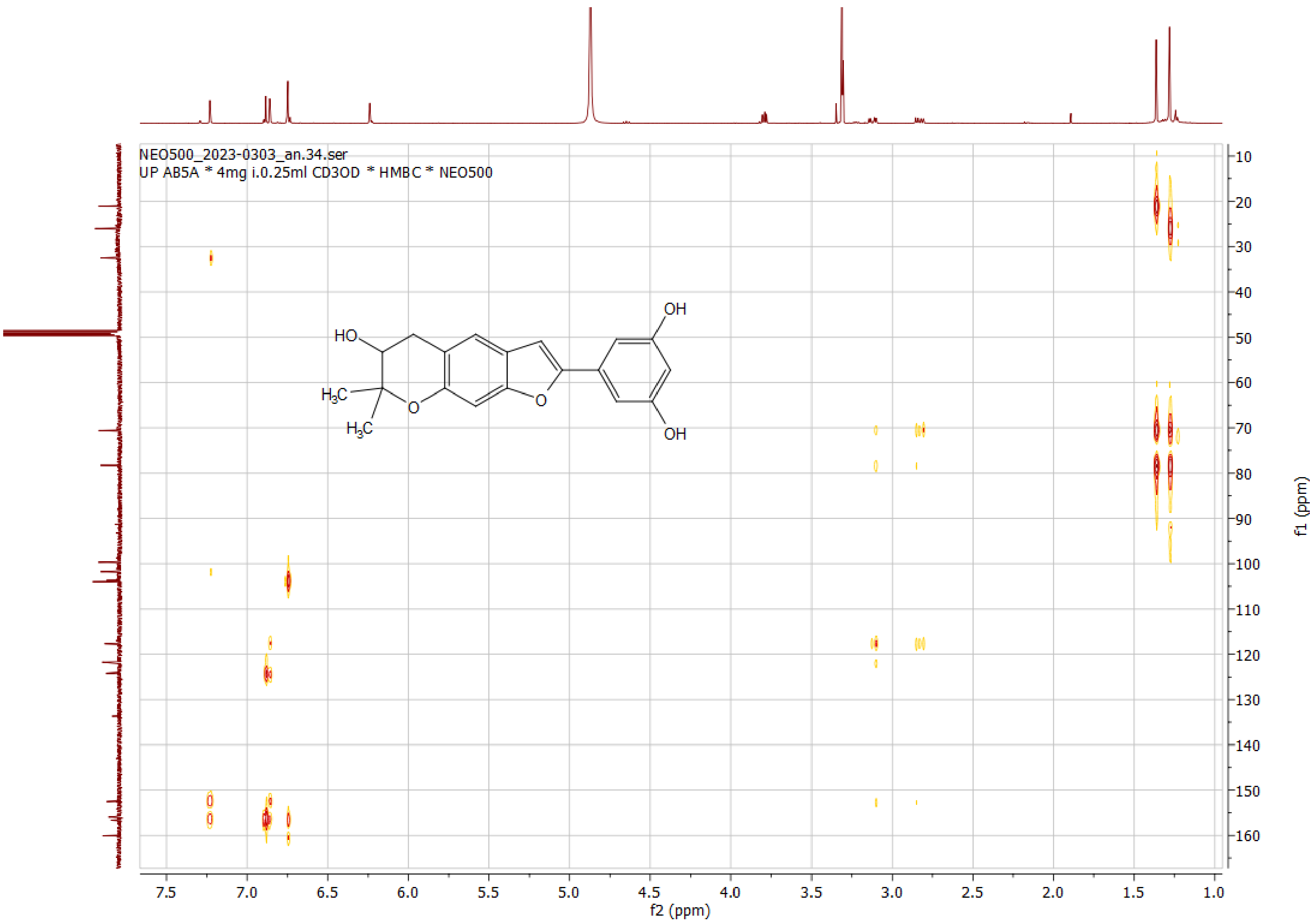

$^{1}$H NMR (500 MHz, CD3OD) of moracin M (**3**)

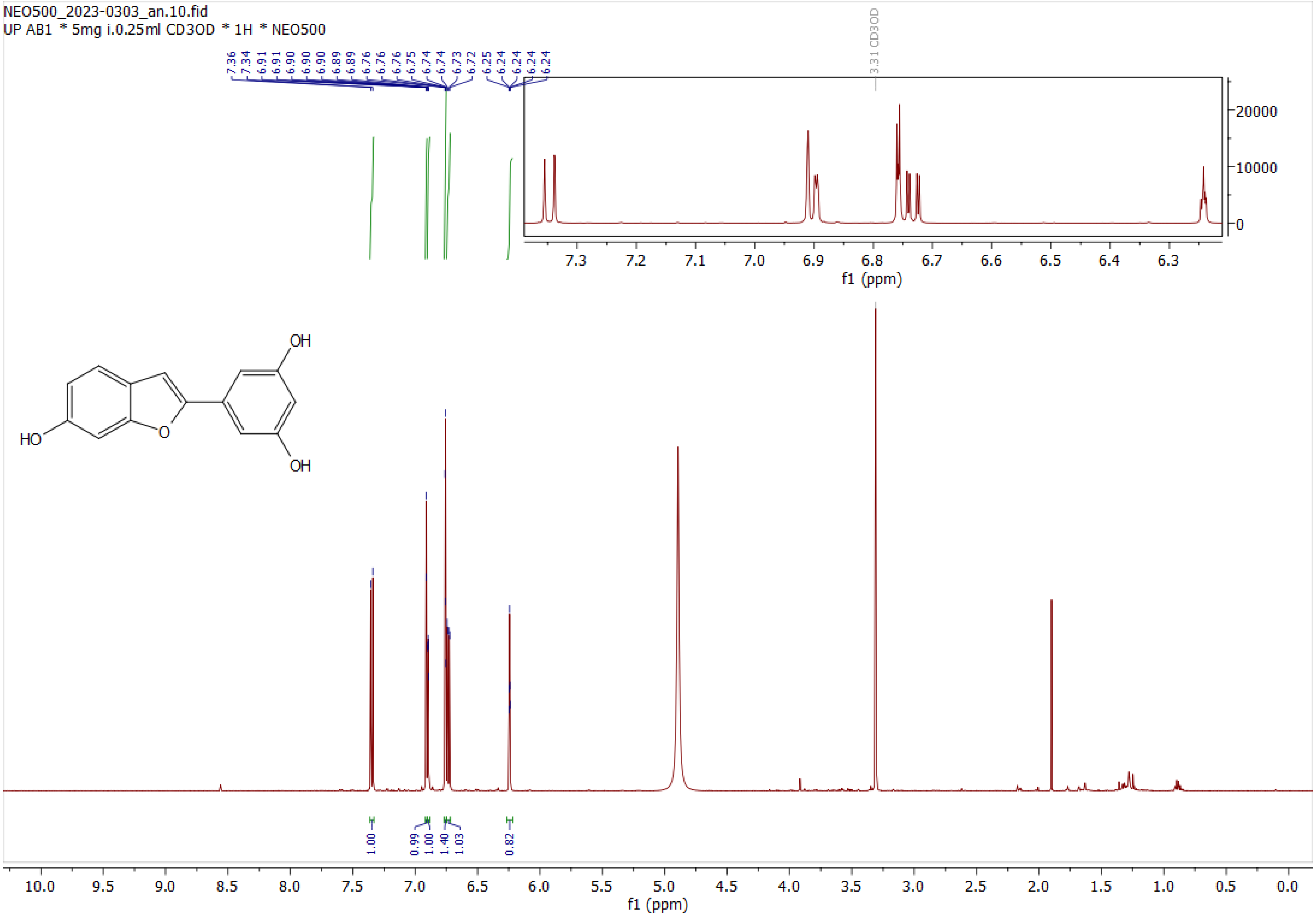

$^{13}C$ NMR (125 MHz, $CD_3OD$) of moracin M (**3**)

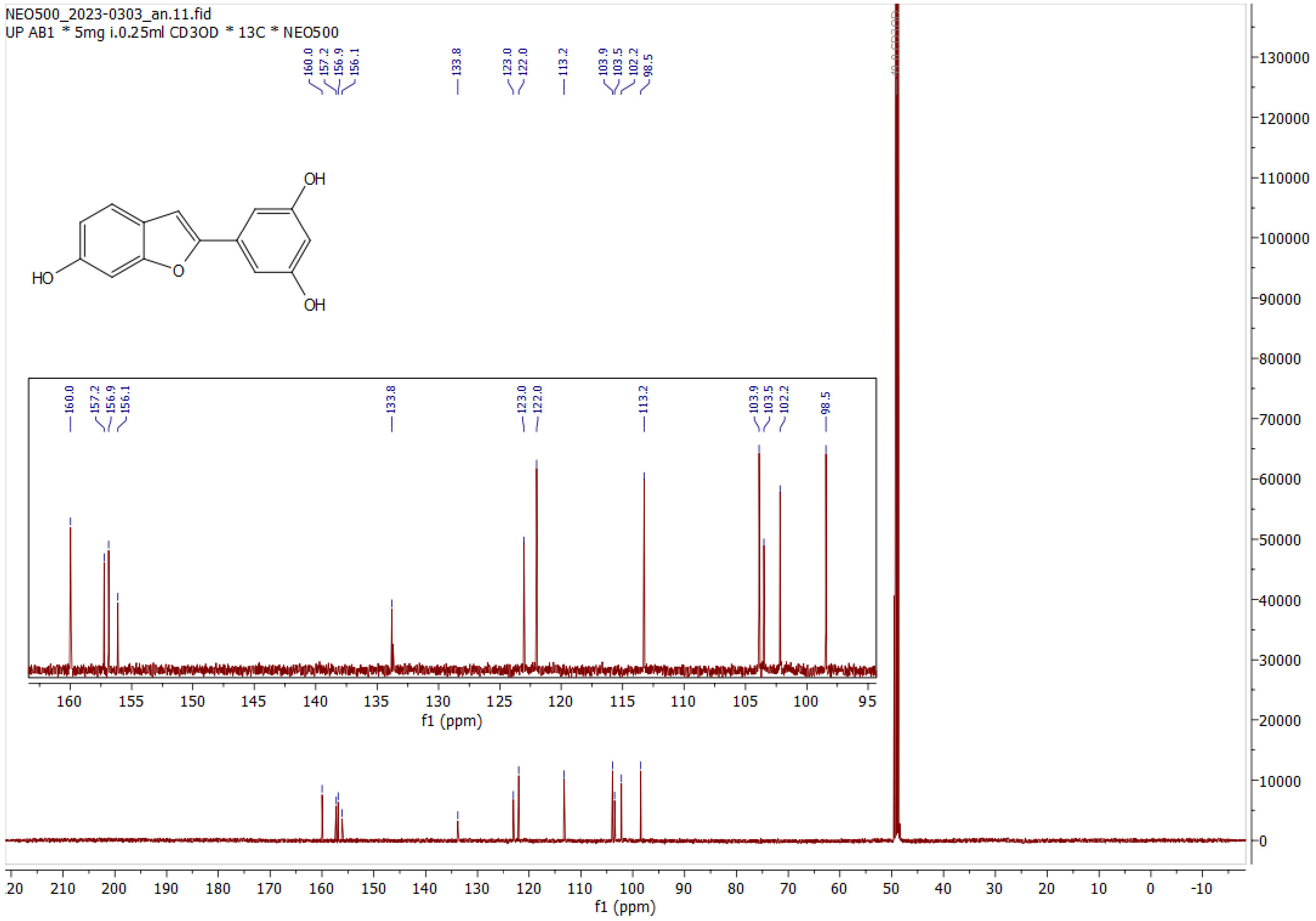

COSY (500 MHz, $CD_3OD$) of moracin M (**3**)

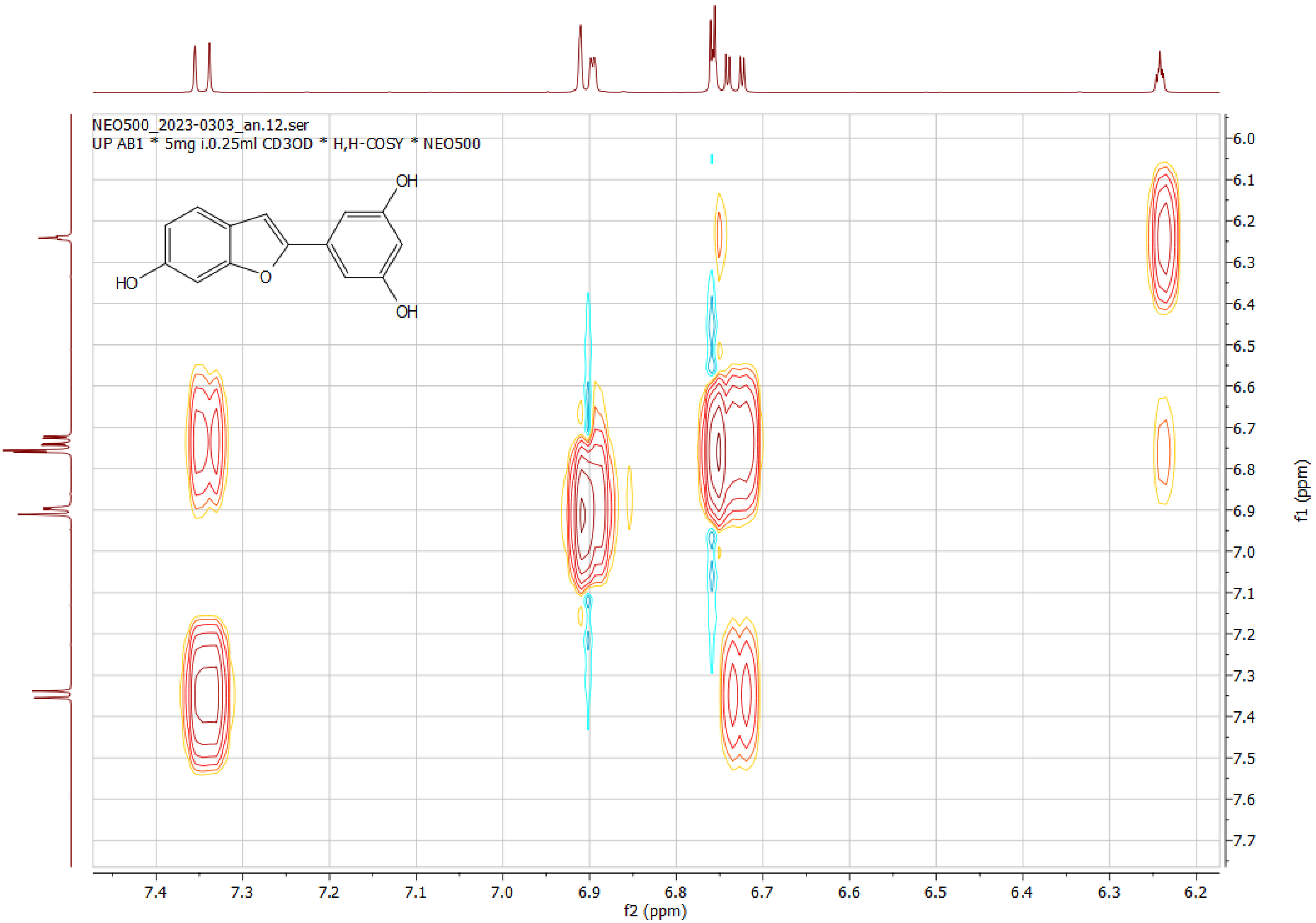

HSQC (500/125 MHz, $CD_3OD$) of moracin M (**3**)

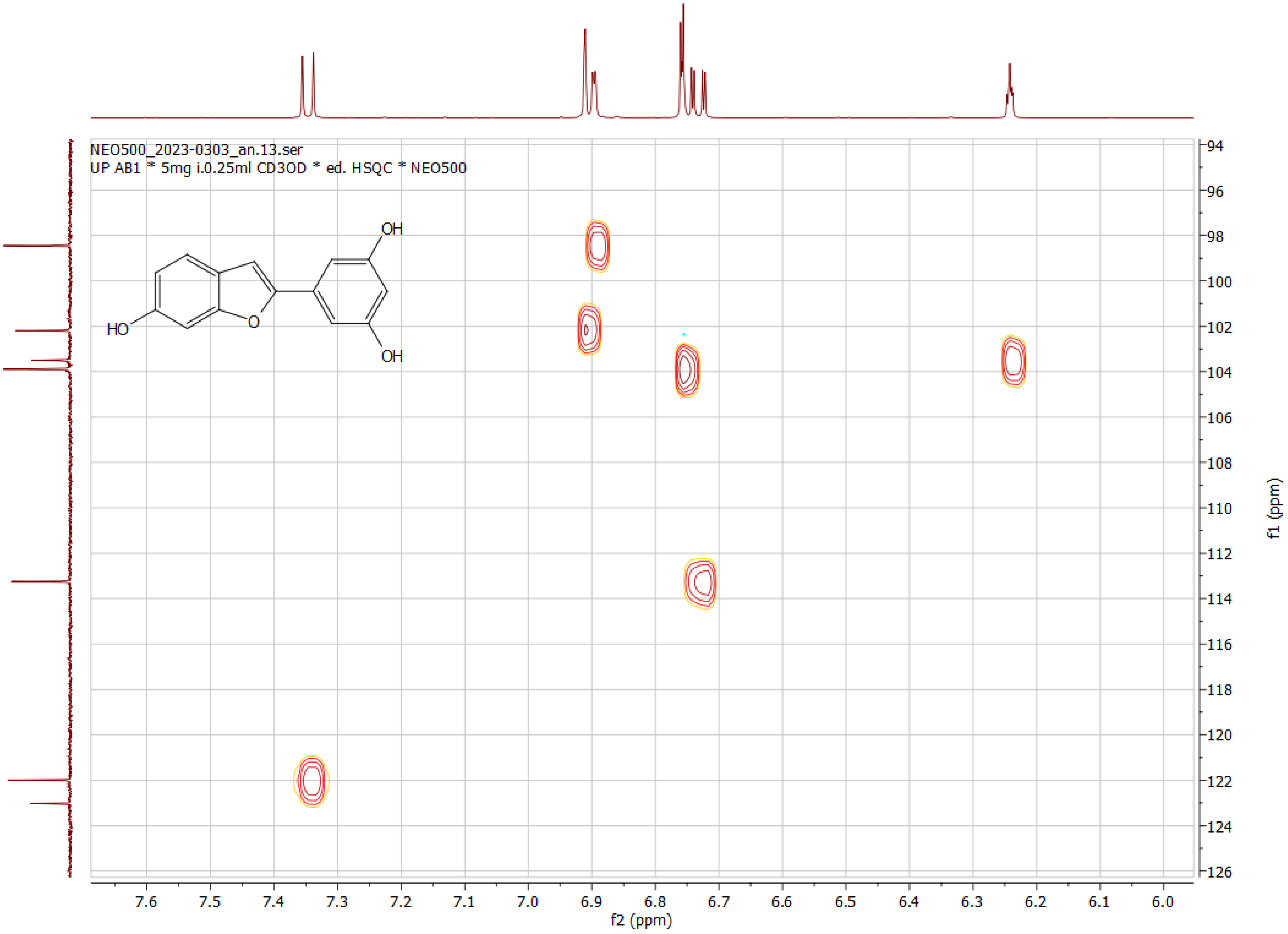

HMBC (500/125 MHz, $CD_3OD$) of moracin M (**3**)

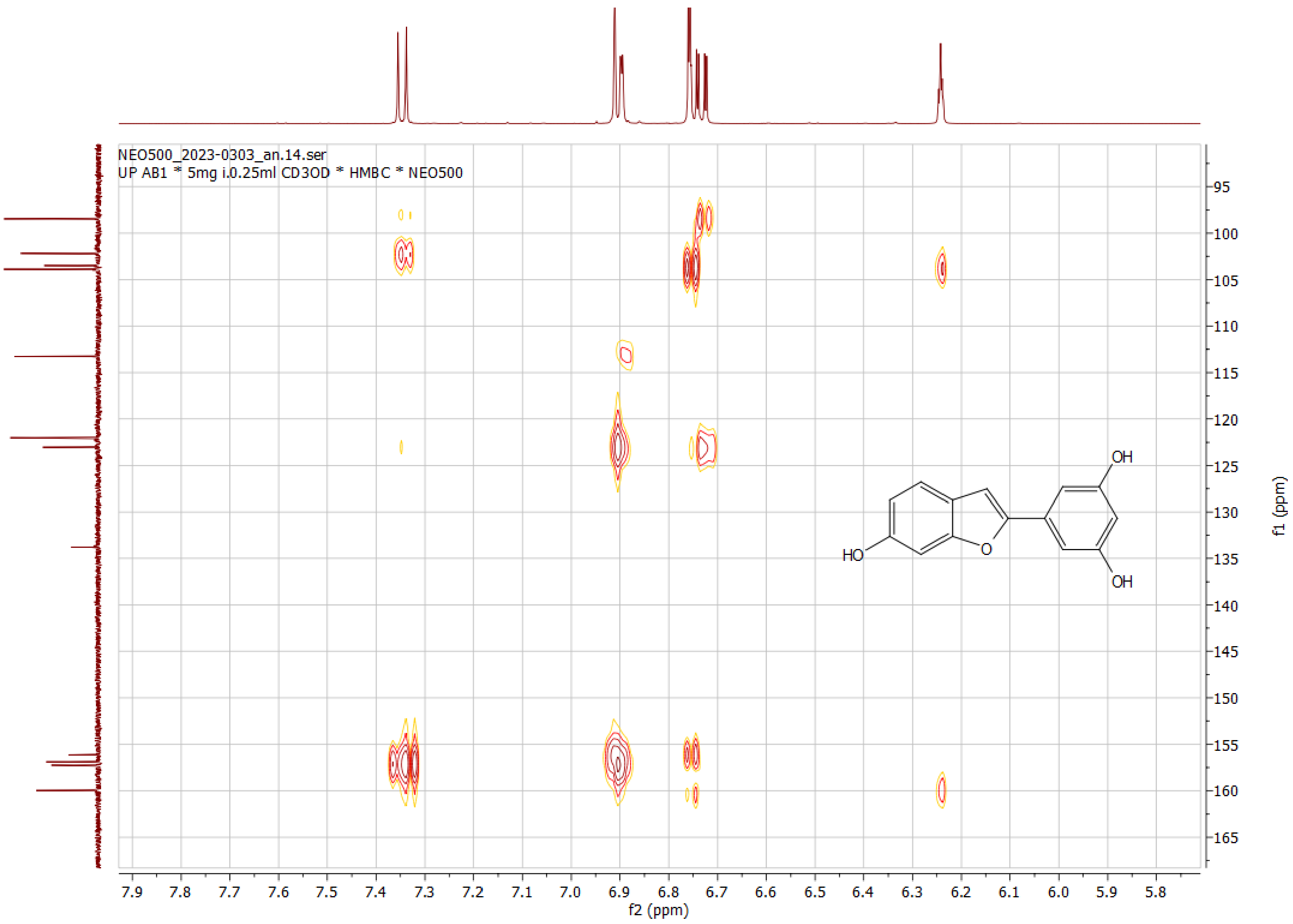

Supplement: Supplementary file 1 [file DataSheet1.PDF]
